# Changes in rural China’s caregiver outcomes, behaviours, and health services utilisation following the COVID-19 pandemic: an observational study

**DOI:** 10.7189/jogh.15.04203

**Published:** 2025-09-26

**Authors:** Isabel Z Wang, Gary L Darmstadt, Andrew Rule, Sarah-Eve Dill, Yunwei Chen, Huan Zhou, Yuju Wu, Scott Rozelle

**Affiliations:** 1 Stanford Center on China’s Economy and Institutions, Stanford University Freeman Spogli Institute for International Studies, Stanford, California, USA; 2 Department of Pediatrics, Stanford University School of Medicine, Stanford, California, USA; 3 West China School of Public Health, Sichuan University, Chengdu, China

## Abstract

**Background:**

Under-resourced communities in rural China have long faced limitations in accessing and utilising caregiver and child healthcare (CCH). The COVID-19 pandemic exacerbated health inequities globally, while its precise impacts on CCH remain understudied. We report differences in parental migration, maternal mental health, household and nutrition expenditures, child feeding practices, and prenatal, postnatal, and childbirth care following pandemic lockdowns in rural China.

**Methods:**

We compared two groups of families with children who grew to the age of six months either before or during lockdowns. We enrolled eligible households from 80 rural townships, randomly selected from four poverty-designated counties in Sichuan Province, China. We interviewed the control group of primary caregivers in November and December of 2019 (pre-COVID-19), and the case group in May of 2020 (approximately five months into the pandemic). Statistical analyses included *t* tests and linear regressions with adjustments. *P*-values <0.05 were considered statistically significant.

**Results:**

Compared to the control group, the case group presented significantly lower paternal migration and more favourable maternal mental health. Caregiving behaviours (including household and nutrition expenditures) and child feeding practices did not differ, except for higher spending on infant micronutrient supplements. Prenatal health services utilisation, including home visits, was slightly higher, while postnatal services utilisation was lower.

**Conclusions:**

Our findings suggest that many aspects of CCH in rural China were similar or improved during the early pandemic lockdowns. These data highlight the importance of promoting targeted public health interventions, such as mental health support initiatives, accessible perinatal care options, and family-centred education campaigns, in under-resourced communities and during future healthcare crises.

The COVID-19 outbreak led to the implementation of strict lockdown measures to protect population health [1]. China imposed travel restrictions that shut down public transportation and led to widespread business closures and income losses [2]. Consequently, the financial stability of under-resourced workers across China grew increasingly uncertain. This was felt most acutely in rural areas, which have long lagged behind urban centres in economic development [3]. Moreover, hundreds of millions of rural Chinese residents migrate internally to urban centres for work [4], and many had already travelled home for Lunar New Year when lockdowns began. Ineligible for urban-residence unemployment insurance, rural residents experienced almost non-existent employment and challenges in accessing high-quality healthcare [5–7].

The impacts of lockdown burdens on rural residents’ health outcomes remain poorly understood, particularly in caregiver and child healthcare (CCH), which refers to the health and well-being of primary caregivers, typically mothers and grandmothers in China, and children under the age of five years. Decades of research note limitations in healthcare resources, health education, maternal health, and child nutrition in rural China, with treatable diseases accounting for around 20% of infant mortality [8–26]. Understanding how lockdowns may have affected mutable CCH outcomes is crucial for promoting growth, stability, and preventive health in post-COVID China.

Given the documented negative association between parental migration and children’s psychosocial development, the unique trends in labour migration seen during the pandemic may have contributed to changes in rural China’s CCH [27]. When caregivers migrate for work, their higher income could help provide for their children’s resources [12,28], while at home, their support could help improve a partner caregiver’s mental health, which is important for children’s developmental outcomes [29–31]. Moreover, rural China faces a mental health crisis among mothers and grandmothers [32,33], with as many as 23.5% of primary caregivers experiencing depression across 118 villages in the northwest [34]. Surveys conducted in 2020–21 indicate that maternal mental health across China was significantly worse during lockdowns [35–37]. Concurrently, several factors were found to be protective of mental health, including good physical health, access to health information, and familial social support [36,38–41]. Direct empirical evidence is needed to show how these conflicting factors merged to shape CCH at the start of the pandemic.

Breastfeeding pattern is another crucial factor of CCH that may have been influenced by the initial pandemic lockdowns. Exclusive breastfeeding refers to the practice of providing only breast milk as nourishment without additional food and water from complementary feeding. The World Health Organization (WHO) and the United Nations Children’s Fund recommend this method of feeding for children under the age of six months and continued breastfeeding with the addition of complementary foods for children aged six months to two years, as breastfeeding has been widely shown to reduce infant morbidity and mortality [42–44]. Despite this guideline, the prevalence of exclusive breastfeeding pre-pandemic was estimated at less than 30% in rural China, indicating suboptimal breastfeeding and an over-reliance on formula and complementary feeding [45–48]. While mothers in China reported that lockdowns directly impacted income, purchasing, and child feeding practices, it is unclear whether income changes drove families to forego expensive formula and favour breastfeeding [49].

Questions remain about the extent to which rural Chinese caregivers utilised CCH services during the pandemic. The utilisation of perinatal services in rural China was already reported to be insufficient, with around 64–75% of expectant mothers reaching the recommended five prenatal visits before the pandemic, and 9% not receiving any checkups when care was available [50–53]. Furthermore, utilisation of postnatal services was worse, with only around 25–50% of mothers receiving the recommended three postnatal visits [54,55], and many mothers attending postnatal visits only if they or their children fell ill [50]. Pandemic lockdowns altered patients’ preferences for healthcare visits and childbirth location. However, it has not been reported whether families were driven toward public or private hospitals [49]. While novel healthcare tools such as telemedicine became favoured internationally to make visits more accessible, only around 4% of rural Chinese residents were aware of this option, highlighting the continued gaps in CCH experienced by many families [7,56,57].

More than five years since the SARS-CoV-2 virus emerged, there remains a need for more evidence on the associations between strict lockdowns and CCH in rural regions. Our research estimates differences in CCH behaviours and outcomes in rural China during the early stages of the COVID-19 pandemic. To achieve this, we compared two groups of families with children who grew to the age of six months either before or during lockdowns. We examined the relationship between lockdowns and CCH by analysing parental migration and maternal mental health, caregiving behaviours in nutrition expenditures and child feeding practices, and the utilisation of prenatal care, postnatal care, and childbirth services. We provide insights that can be used to guide public health interventions surrounding CCH in lockdowns, both in rural China and in under-resourced communities globally.

## METHODS

### Study location and sampling

We collected data from participants selected from four poverty-designated counties in a prefecture of the primarily rural Sichuan Province in western China in two survey waves in 2019 and 2020 [58,59]. We retrieved our study sample from a randomised controlled trial that used multi-stage random cluster sampling, preregistered under International Standard Randomised Controlled Trial Number 16800789. After excluding non-rural townships and those with populations under 10 000 people, we selected 20 townships in each of the four counties using stratified random sampling. In each township, we enrolled 25 households that included either a pregnant woman in the second or third trimester or a child under six months of age. The list of eligible households was provided by local governmental township health centres that supervised pregnant women and infants in their catchment areas. Three of the four counties were located in remote areas with low population density, so few townships had more than 25 households. On average, we selected 13 households per township. However, in those with fewer than 25 eligible households, we selected all of them, and, when necessary, expanded the township area to include villages up to 60 minutes away. In total, 1302 eligible households participated in the first survey wave in 2019, of which 1173 also participated in the second wave in 2020.

To determine associations with the COVID-19 pandemic, we defined two distinct groups based on the timing of the first and second surveys. The ‛pre-COVID-19’ control group included 829 eligible households across 80 rural townships, with children under six months of age at the time of the initial survey in 2019. The ‛COVID-19’ case group included 281 eligible households across 80 rural townships, comprising families with children under six months of age at the time of the second survey in 2020. These designations allowed for CCH comparisons between families that took care of children before or during the pandemic and related lockdowns. Since we collected data from a preregistered randomised controlled trial, we described the sample size calculation in the associated online registry [60], and utilised STROBE cross-sectional reporting guidelines [61].

### Data collection

The research team conducted the first survey with the pre-COVID-19 group during November and December of 2019, around one month before the onset of the COVID-19 pandemic and related strict lockdowns in China. One member of each household, preferably the primary caregiver, was surveyed one-on-one and in person by a trained interviewer for two hours. The questions were previously piloted, and difficult-to-comprehend or sensitive questions had been removed. The second survey was conducted in May 2020 with COVID-19 group, around two months after strict lockdowns began to loosen in China. Follow-up surveys were conducted through telephone calls by trained interviewers, due to concerns for the health and safety of participating families and interviewers amidst the pandemic. Due to the difficulty of implementing full surveys via calls, the second survey instruments were shorter. Importantly, the analyses in this study were only conducted on questions included in both surveys. An attrition calculation arrived at a follow-up interview rate of 90.3%.

The first section of the surveys evaluated parental migration and maternal mental health. Parental migration was assessed by asking whether each parent was presently living with the child and the age of the child when each parent migrated, if relevant. Maternal mental health was assessed using the 10-item Edinburgh Postnatal Depression Scale (EPDS) [62], which has been translated and validated for use in China [63–65]. Following the EPDS methodology, participants assigned values to 10 statements regarding emotional states experienced over the past week, including the feelings of joy, anxiety, and sadness. Responses to each statement were represented as values from zero to three, with higher scores indicating a greater likelihood of depressive symptoms. The total score, calculated by summing all statements scores, ranged from 0 to 30, with a total score of 13 or higher serving as the standard cutoff to indicate potential depression [66]. In our analyses, we used both EPDS average scores and the number of caregivers above the EPDS cutoff in analyses.

The second section of the surveys focussed on caregiving behaviours over the past month. We assessed expenditures through 10 questions covering expenditures on food, snacks, child micronutrient supplements, formula, alcohol and cigarettes, and gifts, along with questions about the child’s formula consumption level and price of formula, which may influence spending. Expenditures were collected in Chinese yuan renminbi (RMB) currency and averaged at the group level. Information about child feeding practices was determined using the WHO Infant and Young Child Feeding Practices indicators [42]. We coded responses according to whether each family completed age-appropriate breastfeeding. For children to be categorised as age-appropriately breastfed, they must have exclusively received breast milk while under age six months. The category of predominantly breastfed included age-appropriate breastfeeding, as well as breastfeeding with the addition of vitamin and mineral supplements, ritual fluids, water and water-based drinks, and fruit juice. However, children must not have consumed food-based fluids, semi-solid or solid foods, or non-human milks, including formula. To categorise children as having been fed any formula or any breast milk, they must have been fed in the respective manner at least once before six months of age. These categories were not mutually exclusive.

The third section of the surveys focussed on perinatal care utilisation. The prenatal section included questions about whether mothers received any prenatal visits at a hospital or clinic and whether any prenatal visits occurred through a home visit by medical providers. Interviewers asked for the number of prenatal visits, and each response was formatted into a binary variable indicating whether the recommended number of prenatal visits (*i.e.* five or more) was reached. For childbirth care, a question was asked about the type of hospital in which each child was born and answered by accessing the child’s birth certificate. For postnatal care, interviewers asked whether mothers and children had received any medical examination after leaving the hospital post-delivery; whether a medical examination had occurred at home, a village clinic, a township-level hospital, or a county-level hospital or above; and whether mothers and children had received a health checkup within 10 days after being discharged from a hospital. The total number of postnatal checkups after discharge was recorded and similarly formatted into a binary variable indicating whether the recommended number of postnatal visits (*i.e.* three or more) was reached.

The final section of the surveys collected demographic information on caregivers and children, including family household assets, childbirth method, child age, child sex, gestational age in weeks at birth, birth height in centimetres, and birth weight in kilograms. For household assets, interviewers collected information on each household’s access to tap water, a water heater, a washing machine, a computer, the internet, a refrigerator, an air conditioner, a motorbike or electric bike, and a car [67]. An overall household asset index (HAI) was calculated using polychoric principal component analysis standardisation and internally standardised and centred at zero, with positive and negative values indicating relatively higher and lower amounts of assets, respectively. Child age, sex, height, and weight were recorded from the child’s birth certificate. Child age was incorporated as both a continuous variable, to assess the average age of each group, and as a categorical variable, with ages distributed in half-months to adjust the data analyses. Finally, respondents were asked to designate which family member was the primary caregiver.

### Statistical analysis

We conducted statistical analyses using descriptive statistics and *t*-tests. We converted each variable into the appropriate type, such as a binary, continuous, or categorical variable. Factors that combined multiple survey responses, such as the categories of child feeding practices or possibility of maternal depression, were developed into new variables.

For discrete variables such as the number of migrating fathers, we listed each numerical count and calculated the percentage of the control or case group that was represented in each count. For continuous variables, such as household expenditures, we determined the mean (x̄) value and standard deviation (SD) per group, and noted the number of survey responses. We conducted *t*-tests for each variable to compare outcomes between groups.

We then performed linear regressions to adjust for confounding variables. This allowed for determination of significance and direction (*i.e.* whether the value was higher or lower). Each analysis was adjusted for potential confounders of HAI, childbirth method, child age, child sex, birth height, birth weight, and gestational age in weeks at birth. We selected these factors by their capacity to influence study outcomes, as adjusting for these differences may help isolate the effect of the pandemic on the study’s variables of interest from family sociodemographic differences.

We conducted statistical analyses in Stata/MP, version 18.0 (StataCorp LLC, College Station, Texas, USA). We considered *P*-values <0.05 to be statistically significant, and calculated the 95% confidence interval (CI) for each adjusted *P*-value.

## RESULTS

### Characteristics of the pre-COVID-19 control group and COVID-19 case group

Characteristics of the pre-COVID-19 control group and the COVID-19 case group were similar, though we found some significant differences (**Table 1**). The HAI of the COVID-19 group was significantly higher than that of the pre-COVID-19 group, whereas there was no difference when comparing household asset quintiles. On the HAI scale, the small discrepancy between groups indicates that levels of wealth were comparable. The average age of children in the pre-COVID-19 group during the initial survey was 88 days (SD = 53), whereas the average age of children in the COVID-19 group during the second survey was significantly greater at 116 days (SD = 50). While birth height and birth weight were also significantly higher and gestational age significantly lower for children in the COVID-19 group, the real-world differences of five millimetres in height, 73 g in weight, and two days in gestational age do not represent meaningful changes. Finally, the choice of childbirth method was similar between groups.

**Table 1:** Demographic characteristics

Variables	Pre-COVID-19 control group, (n = 829)	COVID-19 case group, (n = 281)	*P*-value
Family characteristics			
*Household asset index, x̄ (SD)*	–0.050 (1.117), (n = 829)	0.107 (1.087), (n = 280)	0.041
Families within given household asset quintile, n (%)			0.130
*Poorest*	176 (21.2)	45 (16.1)	
*Poorer*	186 (22.4)	56 (20.0)	
*Middle*	175 (21.1)	58 (20.7)	
*Richer*	173 (20.9)	74 (26.4)	
*Richest*	119 (14.4)	47 (16.8)	
Child characteristics			
*Child age at interview in days, x̄ (SD)*	88 (53), (n = 829)	116 (50), (n = 280)	<0.001
Child age at interview in given categories in months, n (%)			<0.001
*<0.5*	57 (6.9)	7 (2.5)	
*0.5–1*	94 (11.3)	14 (5.0)	
*1–1.5*	73 (8.8)	16 (5.7)	
*1.5–2*	79 (9.5)	12 (4.3)	
*2–2.5*	70 (8.4)	19 (6.8)	
*2.5–3*	70 (8.4)	20 (7.1)	
*3–3.5*	60 (7.2)	18 (6.4)	
*3.5–4*	68 (8.2)	28 (10.0)	
*4–4.5*	65 (7.8)	18 (6.4)	
*4.5–5*	60 (7.2)	33 (11.8)	
*5–5.5*	57 (6.9)	47 (16.8)	
*5.5–6*	76 (9.2)	48 (17.1)	
Birth sex in number of males, n (%)	436 (53.3)	144 (51.4)	0.588
Gestational age at birth in weeks, x̄ (SD)	38.9 (1.8), (n = 827)	38.6 (2.4), (n = 275)	0.035
Birth height in centimetres, x̄ (SD)	49.7 (2.2), (n = 782)	50.2 (5.7), (n = 251)	0.033
Birth weight in grams, x̄ (SD)	3233 (448), (n = 829)	3306 (466), (n = 276)	0.022
Children delivered with given childbirth method, n (%)			0.341
*Natural vaginal birth*	352 (42.5)	115 (42.0)	
*Assisted vaginal birth*	12 (1.4)	1 (0.4)	
*Caesarean section*	465 (56.1)	158 (57.7)	

SD – standard deviation, x̄ – mean

### Associations between the COVID-19 pandemic and caregiver outcomes

Paternal migration was significantly lower at 46.0% prevalence in the COVID-19 group compared with 55.5% in the pre-COVID-19 group (**Table 2**). Additionally, the average age of the child when paternal migration occurred was significantly greater at 1.8 months (SD = 1.3) for the COVID-19 group compared with 1.0 months (SD = 1.2) for the Pre-COVID-19 group. The prevalence of maternal migration remained constant, with minimal maternal migration in both groups (1.1% for COVID-19 and 2.8% for pre-COVID-19). We found significantly lower scores on the EPDS in mothers in the COVID-19 group (x̄ = 3.23, SD = 3.68) compared to the pre-COVID-19 group (x̄ = 5.02, SD = 4.52). Both the continuous EPDS and possible depression indicator, defined as a response sum >13 on the EPDS, revealed that mental health symptoms related to depression were significantly more favourable in the COVID-19 group.

**Table 2:** Caregiver outcomes

Variables	Pre-COVID-19 control group, (n = 829)	COVID-19 case group, (n = 281)	*P*-value	Adjusted *P*-value (adjusted 95% CI)*
Parental migration				
*Families with the mother migrating, n (%)*	23 (2.8)	3 (1.1)	0.101	0.134 (–0.041, 0.005)
*Child age in months when the mother leaves if applicable, x̄ (SD)*	1.7 (1.5), (n = 23)	3.3 (0.6), (n = 3)	0.088	Analysis was not applicable due to few event occurrences
*Families with the father migrating, n (%)*	454 (55.5)	128 (46.0)	0.006	0.033 (–0.164, –0.007**)**
*Child age in months when the father leaves if applicable, x̄ (SD)*	1.0 (1.2), (n = 453)	1.8 (1.3), (n = 128)	<0.001	<0.001 (0.288, 0.911)
Maternal mental health				
*EPDS Value, x̄ (SD)*	5.02 (4.52), (n = 793)	3.23 (3.68), (n = 267)	<0.001	<0.001 (–2.524, –1.102**)**
*Mothers with possible depression according to EPDS, n (%)*	58 (8.0%)	9 (3.5%)	0.012	0.003 (–0.104, –0.021)

EPDS – Edinburgh Postnatal Depression Scale, CI – confidence interval, SD – standard deviation, x̄ – mean

*Adjustments included household asset index, child age (in categories), child sex, birth height, birth weight, gestational age in weeks at birth, and childbirth method.

### Associations between the COVID-19 pandemic and caregiving behaviours

For household and nutrition expenditures, there were no significant differences in spending on food, snacks, alcohol and cigarettes, gifts, or formula between groups (**Table 3**). Reported formula price and the related measure of formula consumption similarly showed no significant differences. However, the amount of spending on child micronutrient supplements was significantly higher for the COVID-19 group (x̄ = RMB 168, SD = 404) compared with the pre-COVID-19 group (x̄ = RMB 81, SD = 238), even after adjusting for demographic characteristics. In feeding practices for children under the age of six months, there were no significant differences between groups in age-appropriate breastfeeding, predominant breastfeeding, prevalence of formula feeding, or prevalence of breastfeeding.

**Table 3:** Caregiving behaviours

Variables	Pre-COVID-19 control group, (n = 829)	COVID-19 case group, (n = 281)	*P*-value	Adjusted *P*-value (adjusted 95% CI)*
Household and nutrition expenditures				
*Spending on food in the past month in RMB, x̄ (SD)*	1296 (942), (n = 713)	1234 (892), (n = 225)	0.386	0.912 (–156.623, 175.325)
*Spending on snacks in the past month in RMB, x̄ (SD)*	30 (252), (n = 826)	17 (84), (n = 272)	0.415	0.317 (–57.909, 18.795)
*Spending on child micronutrient supplements in the past month in RMB, x̄ (SD)*	81 (238), (n = 826)	168 (404), (n = 272)	<0.001	0.048 (0.375, 96.261)
*Spending on alcohol and cigarettes in the past month in RMB, x̄ (SD)*	531 (491), (n = 298)	572 (499), (n = 78)	0.510	0.414 (–260.143, 107.422)
*Spending on gifts in the past month in RMB, x̄ (SD)*	989 (681), (n = 258)	992 (711), (n = 53)	0.980	0.672 (–407.385, 263.440)
*Formula consumption in the past month in cans, x̄ (SD)*	2.6 (1.8), (n = 347)	2.9 (2.2), (n = 118)	0.126	1.000 (–0.499, 0.499)
*Reported formula price in the past month in RMB, x̄ (SD)*	345 (321), (n = 348)	321 (101), (n = 121)	0.408	0.721 (–90.516, 62.729)
*Spending on formula in the past month in RMB, x̄ (SD)*	944 (778), (n = 347)	1186 (1092), (n = 118)	0.009	0.985 (–243.963, 248.681)
Child feeding practices				
*Children age-appropriately breastfed, n (%)*	298 (35.9)	82 (29.3)	0.042	0.226 (–0.123, 0.029)
*Children predominantly breastfed, n (%)*	451 (54.4)	136 (48.6)	0.091	0.495 (–0.106, 0.051)
*Children fed any formula, n (%)*	321 (38.7)	113 (40.4)	0.628	0.661 (–0.061, 0.097)
*Children fed any breast milk, n (%)*	706 (85.2)	225 (80.4)	0.058	0.978 (–0.056, 0.057)

CI – confidence interval, SD – standard deviation, x̄ – mean

*Adjustments included household asset index, child age (in categories), child sex, birth height, birth weight, gestational age in weeks at birth, and childbirth method.

### Associations between the COVID-19 pandemic and caregiver and child healthcare services utilisation

The average number of prenatal health visits was significantly higher for the COVID-19 group (x̄ = 11.4, SD = 5.6) than the pre-COVID-19 group (x̄ = 10.5, SD = 4.0) (**Table 4**). However, even among the pre-COVID-19 group, the average number of prenatal visits was significantly above the recommended guideline of at least five visits, and the likelihood of reaching this guideline was approximately the same (95.0% for COVID-19 and 95.1% for pre-COVID-19) in both groups. The prevalence of attending any prenatal visit at a hospital or clinic was significantly higher in the COVID-19 group (98.5%) than the pre-COVID-19 group (95.5%) before, but not after adjusting for demographic characteristics. The prevalence of any home visit for medical care during pregnancy was significantly greater for the COVID-19 group (49.8% compared to 27.4%).

**Table 4:** Health services utilisation*

Variables	Pre-COVID-19 control group, (n = 829)	COVID-19 case group, (n = 281)	*P*-value	Adjusted *P*-value (adjusted 95% CI)†
Prenatal care				
*Prenatal visits per family, x̄ (SD)*	10.5 (4.0), (n = 737)	11.4 (5.6), (n = 255)	0.003	0.013 (0.205, 1.728)
*Families reaching the recommended five prenatal visits*	788 (95.1)	267 (95.0)	0.981	0.241 (–0.055, 0.014)
*Families that had any prenatal visit at a hospital or clinic*	759 (95.5)	269 (98.5)	0.021	0.205 (–0.011, 0.051)
*Families that had any home visit by medical providers during pregnancy*	218 (27.4)	140 (49.8)	<0.001	<0.001 (0.123, 0.273)
Postnatal care				
*Checkups per family after the child was discharged from the hospital, x̄ (SD)*	2.6 (2.4), (n = 822)	2.2 (1.4), (n = 272)	0.010	<0.001 (–1.285, –0.590)
*Families reaching the recommended three postnatal visits*	349 (42.1)	115 (40.9)	0.730	<0.001 (–0.219, –0.072)
*Families that had any medical examination after childbirth and leaving the hospital*	466 (58.7)	156 (57.4)	0.699	0.006 (–0.186, –0.031)
*Families that had their postnatal examination at home*	74 (15.9)	48 (30.8)	<0.001	<0.001 (0.087, 0.273)
*Families that had their examination at a village clinic*	1 (0.2)	3 (1.9)	0.021	0.063 (–0.001, 0.026)
*Families that had their examination at a township-level hospital*	137 (29.4)	50 (32.1)	0.532	0.676 (–0.130, 0.085)
*Families that had their examination at a county-level hospital*	309 (66.3)	80 (51.3)	<0.001	0.003(–0.282, –0.056)
*Families that received a health check-up within 10 days of being discharged*	535 (65.3)	122 (47.7)	<0.001	<0.001 (–0.285, –0.123)
Childbirth care				
Children delivered at given childbirth location			0.372	0.063 (–0.396, 0.011)
*Home*	0 (0.0)	1 (0.4)		
*Private clinic*	4 (0.5)	2 (0.7)		
*Township health centre*	108 (13.1)	32 (11.6)		
*County (district) maternal and child health hospital*	283 (34.2)	95 (34.5)		
*County (district) hospital*	240 (29.0)	92 (33.5)		
*Municipal (city) maternal and child health hospital*	46 (5.6)	14 (5.1)		
*Municipal (city) hospital*	146 (17.7)	39 (14.2)		

CI – confidence interval, SD – standard deviation, x̄ – mean

*Presented as n (%) unless specified otherwise.

†Adjustments included household asset index, child age (in categories), child sex, birth height, birth weight, gestational age in weeks at birth, and childbirth method.

The average number of postnatal visits was significantly lower for the COVID-19 group (x̄ = 2.2, SD = 1.4) than for the pre-COVID-19 group (x̄ = 2.6, SD = 2.4). In addition, unlike prenatal visits, the likelihood of the COVID-19 group families reaching the recommended guideline of attending at least three postnatal visits was significantly lower (40.9%) than that of the pre-COVID-19 group families (42.1%), and even the pre-COVID-19 group, on average, did not reach the recommended three postnatal visits. Similarly, the prevalences of receiving a medical examination after childbirth and leaving the hospital (57.4% compared to 58.7%), as well as receiving a health check-up within 10 days of being discharged (47.7% compared to 65.3%), were significantly lower for the COVID-19 group compared with the pre-COVID-19 group. The prevalence of receiving a postnatal medical examination at home was significantly greater for the COVID-19 group (30.8% compared to 15.9%), whereas the prevalence of receiving an examination at a county-level hospital was significantly greater for the pre-COVID-19 group (66.3%) compared with the COVID-19 group (51.3%). Childbirth location did not differ significantly between both groups.

## DISCUSSION

Our study provides some of the earliest evidence that many aspects of CCH conditions were similar or improved during the early COVID-19 pandemic in rural China. Paternal migration was lower, and maternal mental health indicators were significantly more favourable in the COVID-19 group. In addition, despite potential losses of income for many families, child feeding expenditures were similar or increased. The most notable negative finding related to CCH was a decline in postnatal care visits.

While the pandemic was associated with an expected significant decrease in paternal migration, the proportion of fathers absent from home remained high for the COVID-19 group (46.0%). However, children were significantly older, by 0.8 months on average, at the time of their fathers’ migration. Perhaps these differences can be attributed to severe lockdowns being lifted slightly before the second survey, with lockdowns still halting travel in the initial months post-childbirth [68]. Compared to paternal migration, there were no significant differences in maternal migration. Consistent with past findings from rural China, many fewer mothers than fathers migrated in either group [69].

Unexpectedly, we found that maternal mental health in rural China was significantly better among the COVID-19 group, in contrast with global reports of heavier burdens on maternal mental health, particularly stress, anxiety, and depressive symptoms [70]. We theorise that fathers staying home during early child development may have contributed to heightened social support, which is associated with improved caregiver mental health [29,30,32,34]. This factor may have outweighed other influences on mental health, and it may play a key role in promoting CCH in policy interventions and responses to future lockdown-like situations [36,39,40,71–75].

We observed that household and nutrition expenditures were similar between groups despite the loss of steady income experienced by many rural migrant workers [76]. Beyond significantly greater spending on child micronutrient supplements in the COVID-19 group compared to the pre-COVID-19 group, any change in income level during this period did not alter spending habits. One international study found that families adjusted expenditures during the pandemic to offset lower income and rising food costs, though these actions did not guarantee food security [77]. However, the pandemic may not have led to major changes in food security or dietary quality in rural China [78]. We posit that the stability of expenditures could reflect that the families we interviewed did not spend beyond necessities pre-pandemic, leaving little room for fluctuation. The stability of gift-giving expenditures also reflects this theme, as strong sociocultural norms in rural China may mandate spending on gifts, despite circumstances [79]. The question remains as to whether spending on healthcare services and resources was considered necessary or auxiliary. Interestingly, spending on alcohol and cigarettes did not change, though an increase from stress-related spending or a decrease from pre-emptive income shock protection may have been expected. This could indicate that use of these substances is a stable feature, and perhaps health counselling or alcohol and tobacco cessation programs would support CCH.

While overall nutrition expenditures remained similar, the COVID-19 group spent significantly more than the pre-COVID-19 group on child micronutrient supplements – most likely the nutrient-dense complementary food supplement Ying Yang Bao [80]. As children were under age six months, the recommended age at which to begin providing Ying Yang Bao, this observed spending increase is surprising [81]. Furthermore, Ying Yang Bao is distributed through a national public health program in rural China, including this study’s region, at no charge [82]. We suggest that COVID-19 prevention policies may have limited access to Ying Yang Bao distribution sites, and COVID-19 group caregivers may have anticipated this change and spent more to ensure it would be available when their child needed it.

In child feeding practices, we found that the balance of breastfeeding and formula feeding was unchanged, though we expected lower rates of formula feeding for the COVID-19 group in relation to widespread income loss [76]. Existing literature on rural China reinforces that childbirth occurring during the pandemic, as well as receiving support from friends or relatives, predicted children being mostly breastfed [49]. Instead, our analyses demonstrated a lack of change – including a slight but non-significant increase in the prevalence of formula feeding, potentially explained by the documented fear of transmitting COVID-19 through breastfeeding – overall indicating that key factors influence families’ nutrition expenditures beyond economic circumstances [83]. In either group, less than 40% of children were age-appropriately breastfed and around 40% were fed formula. Further exploration into the reasons behind current breastfeeding practices in rural China is needed, as these findings may have broader socioeconomic and developmental implications. If the strong use of formula under age six months is due to a lack of either time to breastfeed or social support, then CCH interventions should place greater emphasis on advocating for healthy workplace policies and home environments, as well as educating families about the importance of breastfeeding in children’s early development.

Finally, our results revealed adequate prenatal but insufficient postnatal care utilisation, especially in the COVID-19 group, supporting evidence that the pandemic disrupted CCH services in China and under-resourced communities, such as those in low- and middle-income countries [84–87]. Although the COVID-19 group had a significantly greater number of prenatal visits (11.4 for COVID-19 and 10.5 for pre-COVID-19 group), this was a minor finding, as both groups exceeded the recommended minimum of five prenatal visits in national and global guidelines [88,89]. The lack of group differences may relate to pregnancy stage, as many COVID-19 group women were pregnant pre-lockdown. Since study participants were identified via local governmental township health centres that supervised all pregnant women and infants in their catchment areas, and while coverage should be comprehensive, health services utilisation may have been higher than average. However, postnatal care was significantly worse for the COVID-19 group on multiple levels, indicating that some healthcare services suffered. While the deficit was more severe for the COVID-19 group, the average number of postnatal visits for both groups (2.2 for COVID-19 and 2.6 for pre-COVID-19) fell below the minimum three postnatal visits recommended by the WHO [90]. In addition, the prevalence of receiving a health checkup within 10 days of being discharged was significantly lower in the COVID-19 group (47.7% compared to 65.3%). Our findings also suggest that perinatal visits at home were more common for the COVID-19 group, presumably due to pandemic accommodations, whereas postnatal examination at a county-level hospital was less common. Childbirth locations remained unchanged, as a large majority of mothers chose to give birth at county-level hospitals in both groups. Overall, the underutilisation of postnatal health services during a time of crisis raises concerns for the long-term health of rural and under-resourced communities.

We acknowledge several limitations of the present study. First, as this was an observational study, causality could not be established. We noted significant group differences, but the underlying causes and directionality remain unclear. Second, we lacked qualitative data on caregivers’ decision-making, including reasons for feeding and spending habits. Third, the sample population may not represent families who declined participation or lacked access to local governmental township health centre touchpoints. Fourth, the study sample consisted of fewer pregnant women than those with children, potentially due to rural migration patterns, resulting in a smaller COVID-19 group. The pandemic also prevented longitudinal comparisons of children’s developmental outcomes, as the follow-up interviews were condensed and telephone-based, which may have introduced response bias from reduced survey length and lack of in-person communication. The attrition report notes that around 9.7% of families were lost to follow-up. These study limitations underscore the need for further research on CCH in rural China and the effects of pandemics on under-resourced communities.

## CONCLUSIONS

For vulnerable populations in low- and middle-income settings, including rural Chinese communities, research on CCH is essential for improving health outcomes. Our study provides novel information that paternal but not maternal migration was lower during the COVID-19 pandemic and related lockdowns in rural China; maternal mental health was more favourable; most caregiving behaviours such as household and nutrition expenditures and child feeding practices were no different; and prenatal health services utilisation, including at home, was slightly higher to similar while postnatal health services utilisation was lower. Future studies should investigate the causal links between and qualitative reasons for these findings, as well as report on children’s longitudinal health outcomes following lockdowns. The association between caregiver social support and mental health is especially notable, as mental health and physical health influence one another bidirectionally. Studying the effects of perinatal health education interventions, including those conducted through digital communication platforms, on caregiver attitudes toward exclusive breastfeeding and postnatal care, may be particularly beneficial in supporting children’s healthy development. Our findings demonstrate that targeted public health interventions, in the form of mental health campaigns to expand social support networks for primary caregivers, family-centred education modules on children’s feeding and development, and improved perinatal care outreach initiatives, such as accessible telemedicine appointments, may help support families in rural China and inform the design of improved CCH policies throughout China and beyond.

## Additional material


Online Supplementary Document
