# Supplementary material for: Changes in rural China’s caregiver outcomes, behaviours, and health services utilisation following the COVID-19 pandemic: an observational study
Source: J Glob Health. 2025 Sep 26;15:04203. doi: 10.7189/jogh.15.04203 (PMC12467446; doi:10.7189/jogh.15.04203)
Supplement: Online Supplementary Document [file jogh-15-04203-s001.pdf]

Supplement to: Wang IZ, Darmstadt GL, Rule A, Dill SE, Chen Y, Zhou H, Rozelle S. Changes in rural China's caregiver outcomes, behaviours, and health services utilisation following the COVID-19 pandemic: an observational study. J Glob Health. 2025;15:04203.

STROBE CHECKLIST

STROBE Statement—Checklist of items that should be included in reports of ***cross-sectional studies***

	Item No	Recommendation	Page No
**Title and abstract**	1	(*a*) Indicate the study's design with a commonly used term in the title or the abstract	1
		(*b*) Provide in the abstract an informative and balanced summary of what was done and what was found	1–2
**Introduction**			
Background/rationale	2	Explain the scientific background and rationale for the investigation being reported	2–5
Objectives	3	State specific objectives, including any prespecified hypotheses	4–5
**Methods**			
Study design	4	Present key elements of study design early in the paper	5–9
Setting	5	Describe the setting, locations, and relevant dates, including periods of recruitment, exposure, follow-up, and data collection	5–6
Participants	6	(*a*) Give the eligibility criteria, and the sources and methods of selection of participants	5–6
Variables	7	Clearly define all outcomes, exposures, predictors, potential confounders, and effect modifiers. Give diagnostic criteria, if applicable	6–8
Data sources/ measurement	8*	For each variable of interest, give sources of data and details of methods of assessment (measurement). Describe comparability of assessment methods if there is more than one group	6–8
Bias	9	Describe any efforts to address potential sources of bias	9
Study size	10	Explain how the study size was arrived at	6
Quantitative variables	11	Explain how quantitative variables were handled in the analyses. If applicable, describe which groupings were chosen and why	6–8
Statistical methods	12	(*a*) Describe all statistical methods, including those used to control for confounding	8–9

		(*b*) Describe any methods used to examine subgroups and interactions	8–9
		(*c*) Explain how missing data were addressed	8–9
		(*d*) If applicable, describe analytical methods taking account of sampling strategy	8–9
		(*e*) Describe any sensitivity analyses	N/A
**Results**			
Participants	13*	(a) Report numbers of individuals at each stage of study—eg numbers potentially eligible, examined for eligibility, confirmed eligible, included in the study, completing follow-up, and analysed	Tables 1–4
		(b) Give reasons for non-participation at each stage	5–6
		(c) Consider use of a flow diagram	N/A
Descriptive data	14*	(a) Give characteristics of study participants (eg demographic, clinical, social) and information on exposures and potential confounders	5–6, 9
		(b) Indicate number of participants with missing data for each variable of interest	5–6, Tables 1–4
Outcome data	15*	Report numbers of outcome events or summary measures	Tables 1–4
Main results	16	(*a*) Give unadjusted estimates and, if applicable, confounder-adjusted estimates and their precision (eg, 95% confidence interval). Make clear which confounders were adjusted for and why they were included	Tables 1–4
		(*b*) Report category boundaries when continuous variables were categorized	Tables 1–4
		(*c*) If relevant, consider translating estimates of relative risk into absolute risk for a meaningful time period	N/A
Other analyses	17	Report other analyses done—eg analyses of subgroups and interactions, and sensitivity analyses	Adjustment Analyses for Each Variable of Interest
**Discussion**			
Key results	18	Summarise key results with reference to study objectives	9–11
Limitations	19	Discuss limitations of the study, taking into account sources of potential bias or imprecision. Discuss both direction and magnitude of any potential bias	15
Interpretation	20	Give a cautious overall interpretation of results considering objectives, limitations, multiplicity of analyses, results from similar studies, and other relevant evidence	15–16
Generalisability	21	Discuss the generalisability (external validity) of the study results	15–16

**Other information**			
Funding	22	Give the source of funding and the role of the funders for the present study and, if applicable, for the original study on which the present article is based	16

*Give information separately for exposed and unexposed groups.

Note: An Explanation and Elaboration article discusses each checklist item and gives methodological background and published examples of transparent reporting. The STROBE checklist is best used in conjunction with this article (freely available on the Web sites of PLoS Medicine at http://www.plosmedicine.org/, Annals of Internal Medicine at http://www.annals.org/, and Epidemiology at http://www.epidem.com/). Information on the STROBE Initiative is available at www.strobe-statement.org.

ADJUSTMENT ANALYSES FOR EACH VARIABLE OF INTEREST

These tables explore which confounding factors might be contributing to real-world significance. The descriptive statistics of each variable can be found in the main tables of this study. Each variable is compared between the Pre-Covid control group and the Covid case group. The category of "All Covariates" for adjustment combines the separate factors of childbirth method, child age in categories, household asset index, birth sex, gestational age in weeks at birth, birth height, and birth weight.

Caregiver outcomes

Table 1. Parental migration: Number of families with the mother migrating.

**Covariate Adjustments**	**P-Value**	**95% Confidence Interval, Lower Limit**	**95% Confidence Interval, Upper Limit**
Combined Covariates			
All Covariates	0.134	-0.041	0.005
Isolated Covariates			
Child Age in Categories	0.023*	-0.049	-0.004
Household Asset Index	0.204	-0.034	0.007
Birth Sex	0.097	-0.038	0.003
Gestational Age in Weeks at Birth	0.116	-0.038	0.004
Birth Height	0.273	-0.032	0.009
Birth Weight	0.148	-0.036	0.005
Childbirth Method	0.107	-0.038	0.004

*This symbol indicates a p-value <0.05.

Table 2. Parental migration: Number of families with the father migrating

**Covariate Adjustments**	**P-Value**	**95% Confidence Interval, Lower Limit**	**95% Confidence Interval, Upper Limit**
Combined Covariates			
All Covariates	0.033*	-0.164	-0.007
Isolated Covariates			
Child Age in Categories	0.002*	-0.191	-0.042
Household Asset Index	0.018*	-0.148	-0.014
Birth Sex	0.008*	-0.160	-0.024
Gestational Age in Weeks at Birth	0.006*	-0.164	-0.028
Birth Height	0.026*	-0.152	-0.010
Birth Weight	0.010*	-0.158	-0.022
Childbirth Method	0.007*	-0.162	-0.025

*This symbol indicates a p-value <0.05.

Table 3. Parental migration: Mean child age in months when the mother leaves if applicable

**Covariate Adjustments**	**P-Value**	**95% Confidence Interval, Lower Limit**	**95% Confidence Interval, Upper Limit**
Combined Covariates			
All Covariates	Too few cases.	N/A	N/A
Isolated Covariates			
Child Age in Categories	0.651	-5.365	7.365
Household Asset Index	0.175	-0.633	3.280
Birth Sex	0.045*	0.048	3.850
Gestational Age in Weeks at Birth	0.057	-0.057	3.411
Birth Height	0.069	-0.146	3.581
Birth Weight	0.093	-0.259	3.128
Childbirth Method	0.096	-0.306	3.476

*This symbol indicates a p-value <0.05.

Table 4. Parental migration: Mean child age in months when the father leaves if applicable

**Covariate Adjustments**	**P-Value**	**95% Confidence Interval, Lower Limit**	**95% Confidence Interval, Upper Limit**
Combined Covariates			
All Covariates	<0.001*	0.288	0.911
Isolated Covariates			
Child Age in Categories	<0.001*	0.324	0.888
Household Asset Index	<0.001*	0.537	1.027
Birth Sex	<0.001*	0.538	1.028
Gestational Age in Weeks at Birth	<0.001*	0.572	1.065
Birth Height	<0.001*	0.557	1.051
Birth Weight	<0.001*	0.557	1.051
Childbirth Method	<0.001*	0.547	1.039

*This symbol indicates a p-value <0.05.

Table 5. Maternal mental health: Mean EPDS depression scale value

**Covariate Adjustments**	**P-Value**	**95% Confidence Interval, Lower Limit**	**95% Confidence Interval, Upper Limit**
Combined Covariates			
All Covariates	<0.001*	-2.524	-1.102
Isolated Covariates			
Child Age in Categories	<0.001*	-2.437	-1.122
Household Asset Index	<0.001*	-2.390	-1.185
Birth Sex	<0.001*	-2.390	-1.184
Gestational Age in Weeks at Birth	<0.001*	-2.362	-1.150

Birth Height	<0.001*	-2.447	-1.187
Birth Weight	<0.001*	-2.363	-1.150
Childbirth Method	<0.001*	-2.388	-1.177

*This symbol indicates a p-value <0.05.

Table 6. Maternal mental health: Number of mothers with possible depression according to EPDS depression scale

**Covariate Adjustments**	**P-Value**	**95% Confidence Interval, Lower Limit**	**95% Confidence Interval, Upper Limit**
Combined Covariates			
All Covariates	0.003*	-0.104	-0.021
Isolated Covariates			
Child Age in Categories	0.006*	-0.096	-0.016
Household Asset Index	0.012*	-0.082	-0.010
Birth Sex	0.016*	-0.079	-0.008
Gestational Age in Weeks at Birth	0.018*	-0.080	-0.007
Birth Height	0.015*	-0.080	-0.009
Birth Weight	0.011*	-0.083	-0.011
Childbirth Method	0.008*	-0.084	-0.013

*This symbol indicates a p-value <0.05.

Caregiving behaviours

Table 7. Household and nutrition expenditures: Mean spending on food in the past month in RMB

**Covariate Adjustments**	**P-Value**	**95% Confidence Interval, Lower Limit**	**95% Confidence Interval, Upper Limit**
Combined Covariates			
All Covariates	0.912	-156.623	175.325
Isolated Covariates			
Child Age in Categories	0.860	-168.184	140.374
Household Asset Index	0.273	-217.397	61.465
Birth Sex	0.387	-202.661	78.532
Gestational Age in Weeks at Birth	0.372	-205.974	77.211
Birth Height	0.778	-167.660	125.521
Birth Weight	0.381	-203.668	77.827
Childbirth Method	0.305	-213.401	66.850

*This symbol indicates a p-value <0.05.

Table 8. Household and nutrition expenditures: Mean spending on snacks in the past month in RMB

**Covariate Adjustments**	**P-Value**	**95% Confidence Interval, Lower Limit**	**95% Confidence Interval, Upper Limit**
Combined Covariates			
All Covariates	0.317	-57.909	18.795
Isolated Covariates			
Child Age in Categories	0.380	-49.383	18.852
Household Asset Index	0.442	-42.702	18.663
Birth Sex	0.420	-43.466	18.157
Gestational Age in Weeks at Birth	0.430	-43.329	18.470
Birth Height	0.381	-47.940	18.342

Birth Weight	0.380	-44.628	17.044
Childbirth Method	0.408	-43.854	17.824

*This symbol indicates a p-value <0.05.

Table 9. Household and nutrition expenditures: Mean spending on child micronutrient supplements in the past month in RMB

**Covariate Adjustments**	**P-Value**	**95% Confidence Interval, Lower Limit**	**95% Confidence Interval, Upper Limit**
Combined Covariates			
All Covariates	0.048*	0.375	96.261
Isolated Covariates			
Child Age in Categories	0.007*	16.286	105.283
Household Asset Index	<0.001*	44.223	123.283
Birth Sex	<0.001*	47.575	127.283
Gestational Age in Weeks at Birth	<0.001*	46.733	126.545
Birth Height	<0.001*	38.545	121.513
Birth Weight	<0.001*	49.947	129.733
Childbirth Method	<0.001*	49.200	128.647

*This symbol indicates a p-value <0.05.

Table 10. Household and nutrition expenditures: Mean spending on alcohol and cigarettes in the past month in RMB

**Covariate Adjustments**	**P-Value**	**95% Confidence Interval, Lower Limit**	**95% Confidence Interval, Upper Limit**
Combined Covariates			
All Covariates	0.414	-260.143	107.422
Isolated Covariates			

Child Age in Categories	0.585	-203.386	115.091
Household Asset Index	0.717	-98.654	143.269
Birth Sex	0.540	-85.790	163.692
Gestational Age in Weeks at Birth	0.604	-92.533	158.856
Birth Height	0.455	-83.363	185.656
Birth Weight	0.637	-94.678	154.463
Childbirth Method	0.704	-101.107	149.476

*This symbol indicates a p-value <0.05.

Table 11. Household and nutrition expenditures: Mean spending on gifts in the past month in RMB

**Covariate Adjustments**	**P-Value**	**95% Confidence Interval, Lower Limit**	**95% Confidence Interval, Upper Limit**
Combined Covariates			
All Covariates	0.672	-407.385	263.440
Isolated Covariates			
Child Age in Categories	0.968	-278.273	289.941
Household Asset Index	0.966	-201.361	210.353
Birth Sex	0.962	-199.594	209.402
Gestational Age in Weeks at Birth	0.979	-202.283	207.773
Birth Height	0.831	-236.791	190.314
Birth Weight	0.984	-207.526	203.366
Childbirth Method	0.979	-201.174	206.607

*This symbol indicates a p-value <0.05.

Table 12. Household and nutrition expenditures: Mean formula consumption in the past month in cans

**Covariate Adjustments**	**P-Value**	**95% Confidence Interval, Lower Limit**	**95% Confidence Interval, Upper Limit**
Combined Covariates			
All Covariates	1.000	-0.499	0.499
Isolated Covariates			
Child Age in Categories	0.828	-0.496	0.397
Household Asset Index	0.118	-0.080	0.708
Birth Sex	0.134	-0.093	0.694
Gestational Age in Weeks at Birth	0.149	-0.104	0.686
Birth Height	0.164	-0.121	0.715
Birth Weight	0.125	-0.086	0.704
Childbirth Method	0.147	-0.103	0.686

*This symbol indicates a p-value <0.05.

Table 13. Household and nutrition expenditures: Mean reported formula price in the past month in RMB

**Covariate Adjustments**	**P-Value**	**95% Confidence Interval, Lower Limit**	**95% Confidence Interval, Upper Limit**
Combined Covariates			
All Covariates	0.721	-90.516	62.729
Isolated Covariates			
Child Age in Categories	0.605	-81.879	47.776
Household Asset Index	0.515	-77.792	39.027
Birth Sex	0.417	-82.781	34.323
Gestational Age in Weeks at Birth	0.395	-84.051	33.250
Birth Height	0.398	-92.691	36.936

Birth Weight	0.423	-82.762	34.761
Childbirth Method	0.396	-83.926	33.226

*This symbol indicates a p-value <0.05.

Table 14. Household and nutrition expenditures: Mean spending on formula in the past month in RMB

**Covariate Adjustments**	**P-Value**	**95% Confidence Interval, Lower Limit**	**95% Confidence Interval, Upper Limit**
Combined Covariates			
All Covariates	0.985	-243.963	248.681
Isolated Covariates			
Child Age in Categories	0.993	-220.271	218.271
Household Asset Index	0.011*	55.298	421.638
Birth Sex	0.010*	57.525	422.056
Gestational Age in Weeks at Birth	0.010*	58.711	424.132
Birth Height	0.010*	59.630	446.933
Birth Weight	0.007*	69.765	435.906
Childbirth Method	0.009*	59.772	425.319

*This symbol indicates a p-value <0.05.

Table 15. Child feeding practices: Number of children age-appropriately breastfed

**Covariate Adjustments**	**P-Value**	**95% Confidence Interval, Lower Limit**	**95% Confidence Interval, Upper Limit**
Combined Covariates			
All Covariates	0.226	-0.123	0.029
Isolated Covariates			

Child Age in Categories	0.472	-0.096	0.044
Household Asset Index	0.047*	-0.130	-0.001
Birth Sex	0.029*	-0.136	-0.007
Gestational Age in Weeks at Birth	0.048*	-0.131	-0.001
Birth Height	0.029*	-0.144	-0.008
Birth Weight	0.039*	-0.133	-0.004
Childbirth Method	0.055	-0.129	0.001

*This symbol indicates a p-value <0.05.

Table 16. Child feeding practices: Number of children predominantly breastfed

**Covariate Adjustments**	**P-Value**	**95% Confidence Interval, Lower Limit**	**95% Confidence Interval, Upper Limit**
Combined Covariates			
All Covariates	0.495	-0.106	0.051
Isolated Covariates			
Child Age in Categories	0.525	-0.097	0.050
Household Asset Index	0.106	-0.124	0.012
Birth Sex	0.083	-0.128	0.008
Gestational Age in Weeks at Birth	0.121	-0.122	0.014
Birth Height	0.202	-0.117	0.025
Birth Weight	0.090	-0.126	0.010
Childbirth Method	0.144	-0.119	0.017

*This symbol indicates a p-value <0.05.

Table 17. Child feeding practices: Number of children fed any formula

Covariate Adjustments	P-Value	95% Confidence Interval, Lower Limit	95% Confidence Interval, Upper Limit
Combined Covariates			
All Covariates	0.661	-0.061	0.097
Isolated Covariates			
Child Age in Categories	0.886	-0.068	0.079
Household Asset Index	0.697	-0.053	0.080
Birth Sex	0.662	-0.052	0.081
Gestational Age in Weeks at Birth	0.543	-0.046	0.088
Birth Height	0.622	-0.052	0.087
Birth Weight	0.492	-0.043	0.090
Childbirth Method	0.544	-0.046	0.087

*This symbol indicates a p-value <0.05.

Table 18. Child feeding practices: Number of children fed any breast milk

Covariate Adjustments	P-Value	95% Confidence Interval, Lower Limit	95% Confidence Interval, Upper Limit
Combined Covariates			
All Covariates	0.978	-0.056	0.057
Isolated Covariates			
Child Age in Categories	0.977	-0.053	0.054
Household Asset Index	0.064	-0.097	0.003
Birth Sex	0.055	-0.099	0.001
Gestational Age in Weeks at Birth	0.151	-0.086	0.013
Birth Height	0.061	-0.101	0.002
Birth Weight	0.111	-0.090	0.009

Childbirth Method	0.141	-0.087	0.012

*This symbol indicates a p-value <0.05.

Health services utilisation

Table 19. Prenatal care: Mean number of prenatal visits per family

**Covariate Adjustments**	**P-Value**	**95% Confidence Interval, Lower Limit**	**95% Confidence Interval, Upper Limit**
Combined Covariates			
All Covariates	0.013*	0.205	1.728
Isolated Covariates			
Child Age in Categories	0.011*	0.210	1.627
Household Asset Index	0.005*	0.275	1.529
Birth Sex	0.002*	0.358	1.641
Gestational Age in Weeks at Birth	0.002*	0.386	1.674
Birth Height	0.004*	0.310	1.655
Birth Weight	0.003*	0.319	1.604
Childbirth Method	0.003*	0.340	1.622

*This symbol indicates a p-value <0.05.

Table 20. Prenatal care: Number of families reaching the recommended five prenatal visits

**Covariate Adjustments**	**P-Value**	**95% Confidence Interval, Lower Limit**	**95% Confidence Interval, Upper Limit**
Combined Covariates			
All Covariates	0.241	-0.055	0.014
Isolated Covariates			

Child Age in Categories	0.509	-0.044	0.022
Household Asset Index	0.836	-0.033	0.026
Birth Sex	0.975	-0.029	0.030
Gestational Age in Weeks at Birth	0.887	-0.032	0.028
Birth Height	0.674	-0.037	0.024
Birth Weight	0.899	-0.032	0.028
Childbirth Method	0.895	-0.032	0.028

*This symbol indicates a p-value <0.05.

Table 21. Prenatal care: Number of families that had any prenatal visit at a hospital or clinic

**Covariate Adjustments**	**P-Value**	**95% Confidence Interval, Lower Limit**	**95% Confidence Interval, Upper Limit**
Combined Covariates			
All Covariates	0.205	-0.011	0.051
Isolated Covariates			
Child Age in Categories	0.072	-0.002	0.056
Household Asset Index	0.020*	0.005	0.057
Birth Sex	0.021*	0.005	0.057
Gestational Age in Weeks at Birth	0.011*	0.008	0.060
Birth Height	0.030*	0.003	0.057
Birth Weight	0.010*	0.008	0.060
Childbirth Method	0.010*	0.008	0.060

*This symbol indicates a p-value <0.05.

Table 22. Prenatal care: Number of families that had any home visit by medical providers during pregnancy

**Covariate Adjustments**	**P-Value**	**95% Confidence Interval, Lower Limit**	**95% Confidence Interval, Upper Limit**
Combined Covariates			
All Covariates	<0.001*	0.123	0.273
Isolated Covariates			
Child Age in Categories	<0.001*	0.142	0.282
Household Asset Index	<0.001*	0.161	0.287
Birth Sex	<0.001*	0.159	0.285
Gestational Age in Weeks at Birth	<0.001*	0.161	0.288
Birth Height	<0.001*	0.151	0.283
Birth Weight	<0.001*	0.157	0.284
Childbirth Method	<0.001*	0.162	0.289

*This symbol indicates a p-value <0.05.

Table 23. Postnatal care: Mean number of checkups per family after the child Was discharged from the hospital

**Covariate Adjustments**	**P-Value**	**95% Confidence Interval, Lower Limit**	**95% Confidence Interval, Upper Limit**
Combined Covariates			
All Covariates	<0.001*	-1.285	-0.590
Isolated Covariates			
Child Age in Categories	<0.001*	-1.194	-0.555
Household Asset Index	0.008*	-0.708	-0.106
Birth Sex	0.010*	-0.700	-0.096
Gestational Age in Weeks at Birth	0.007*	-0.716	-0.115
Birth Height	0.005*	-0.766	-0.133

Birth Weight	0.013*	-0.681	-0.079
Childbirth Method	0.010*	-0.694	-0.094

*This symbol indicates a p-value <0.05.

Table 24. Postnatal care: Number of families reaching the recommended three postnatal visits

**Covariate Adjustments**	**P-Value**	**95% Confidence Interval, Lower Limit**	**95% Confidence Interval, Upper Limit**
Combined Covariates			
All Covariates	<0.001*	-0.219	-0.072
Isolated Covariates			
Child Age in Categories	0.002*	-0.175	-0.038
Household Asset Index	0.589	-0.085	0.049
Birth Sex	0.717	-0.079	0.055
Gestational Age in Weeks at Birth	0.523	-0.089	0.046
Birth Height	0.210	-0.115	0.025
Birth Weight	0.540	-0.088	0.046
Childbirth Method	0.495	-0.091	0.044

*This symbol indicates a p-value <0.05.

Table 25. Postnatal care: Number of families that had any medical examination after childbirth and leaving the hospital

**Covariate Adjustments**	**P-Value**	**95% Confidence Interval, Lower Limit**	**95% Confidence Interval, Upper Limit**
Combined Covariates			
All Covariates	0.006*	-0.186	-0.031
Isolated Covariates			
Child Age in Categories	0.026*	-0.155	-0.010
Household Asset Index	0.726	-0.080	0.056
Birth Sex	0.642	-0.084	0.056

Gestational Age in Weeks at Birth	0.724	-0.081	0.056
Birth Height	0.481	-0.079	0.058
Birth Weight	0.763	-0.079	0.058
Childbirth Method	0.749	-0.080	0.057

*This symbol indicates a p-value <0.05.

Table 26. Postnatal care: Number of families that had their examination at home

**Covariate Adjustments**	**P-Value**	**95% Confidence Interval, Lower Limit**	**95% Confidence Interval, Upper Limit**
Combined Covariates			
All Covariates	<0.001*	0.087	0.273
Isolated Covariates			
Child Age in Categories	0.003*	0.044	0.213
Household Asset Index	<0.001*	0.079	0.222
Birth Sex	<0.001*	0.079	0.222
Gestational Age in Weeks at Birth	<0.001*	0.079	0.223
Birth Height	<0.001*	0.108	0.259
Birth Weight	<0.001*	0.080	0.223
Childbirth Method	<0.001*	0.081	0.225

*This symbol indicates a p-value <0.05.

Table 27. Postnatal care: Number of families that had their examination at a village clinic

**Covariate Adjustments**	**P-Value**	**95% Confidence Interval, Lower Limit**	**95% Confidence Interval, Upper Limit**
Combined Covariates			

All Covariates	0.063	-0.001	0.026
Isolated Covariates			
Child Age in Categories	0.133	-0.004	0.029
Household Asset Index	0.017*	0.003	0.032
Birth Sex	0.026*	0.002	0.031
Gestational Age in Weeks at Birth	0.003*	0.007	0.032
Birth Height	0.010*	0.003	0.026
Birth Weight	0.021*	0.003	0.032
Childbirth Method	0.020*	0.003	0.032

*This symbol indicates a p-value <0.05.

Table 28. Postnatal care: Number of families that had their examination at a township-level hospital

**Covariate Adjustments**	**P-Value**	**95% Confidence Interval, Lower Limit**	**95% Confidence Interval, Upper Limit**
Combined Covariates			
All Covariates	0.676	-0.130	0.085
Isolated Covariates			
Child Age in Categories	0.740	-0.114	0.081
Household Asset Index	0.498	-0.055	0.112
Birth Sex	0.536	-0.057	0.110
Gestational Age in Weeks at Birth	0.484	-0.054	0.114
Birth Height	0.740	-0.072	0.102
Birth Weight	0.514	-0.056	0.112
Childbirth Method	0.566	-0.059	0.108

*This symbol indicates a p-value <0.05.

Table 29. Postnatal care: Number of families that had their examination at a county-level hospital

Covariate Adjustments	P-Value	95% Confidence Interval, Lower Limit	95% Confidence Interval, Upper Limit
Combined Covariates			
All Covariates	0.003*	-0.282	-0.056
Isolated Covariates			
Child Age in Categories	0.018*	-0.230	-0.022
Household Asset Index	0.001*	-0.241	-0.067
Birth Sex	0.001*	-0.237	-0.062
Gestational Age in Weeks at Birth	<0.001*	-0.244	-0.069
Birth Height	<0.001*	-0.255	-0.073
Birth Weight	0.001*	-0.242	-0.067
Childbirth Method	0.001*	-0.237	-0.062

*This symbol indicates a p-value <0.05.

Table 30. Postnatal care: Number of families that received a health check-up within 10 days of being discharged

Covariate Adjustments	P-Value	95% Confidence Interval, Lower Limit	95% Confidence Interval, Upper Limit
Combined Covariates			
All Covariates	<0.001*	-0.285	-0.123
Isolated Covariates			
Child Age in Categories	<0.001*	-0.260	-0.108
Household Asset Index	<0.001*	-0.248	-0.112
Birth Sex	<0.001*	-0.243	-0.107
Gestational Age in Weeks at Birth	<0.001*	-0.245	-0.109
Birth Height	<0.001*	-0.262	-0.121

Birth Weight	<0.001*	-0.248	-0.112
Childbirth Method	<0.001*	-0.245	-0.109

*This symbol indicates a p-value <0.05.

Table 31. Childbirth care: Number of children delivered at given childbirth location

**Covariate Adjustments**	**P-Value**	**95% Confidence Interval, Lower Limit**	**95% Confidence Interval, Upper Limit**
Combined Covariates			
All Covariates	0.063	-0.396	0.011
Isolated Covariates			
Child Age in Categories	0.224	-0.309	0.072
Household Asset Index	0.343	-0.256	0.089
Birth Sex	0.373	-0.251	0.094
Gestational Age in Weeks at Birth	0.309	-0.261	0.083
Birth Height	0.225	-0.292	0.069
Birth Weight	0.423	-0.243	0.102
Childbirth Method	0.445	-0.239	0.105

*This symbol indicates a p-value <0.05.

ATTRITION REPORT

HF COVID Phone Survey
June 20, 2020

The baseline sample size was 1303, 3 miscarriages and 2 babies died, so the target sample size of the phone survey is 1299.

	n	%
Total number of baseline samples	1303	100.00%
Abortion	3	0.23%
Infant death	2	0.15%
Target	1299	99.69%

The number of interviews completed is 1,173, accounting for 90.3%; the number of lost interviews is 126 (9.7%).

- Among 1173 interviewed samples, 1152 (88.68%) completed the interview completely, and 24 (1.85%) partially completed the interview.
- Of the 126 people who were lost to follow-up, 113 (8.70%) still refused our interviews with the help of doctors; the other 13 people could not be found, and the doctors could not find them.

Target	1299	100.00%
Completed the interview	1173	90.30%
Full completed interviews	1152	88.68%
Partially completed interviews	24	1.85%
Lost interviews	126	9.70%
Still refused, after the doctor's help	113	8.70%
Doctors can't contact them	13	1.00%

The following table reports the missing information from the 24 partially completed surveys. Most of the information are missing after F1

24 Partially Completed Survey

Module	No. of missing
A	0
B	2
C1	2
C2	5
C3	8
D	9
F1	20
F2	20
G	19
J	19

BASELINE SURVEY

健康未来---四川省南充市基线家长问卷

Healthy Future---Baseline Caregiver Survey in Nanchong, Sichuan

表头 **Header** 24

A. 家庭成员基本信息**Household Roster** 25

B. 家庭背景 Family background 27
B1. 家庭信息Household Info 27
B2. 妈妈外出情况Maternal Migration....................29
C. 喂养行为 Feeding Practices....................31
C1. 婴幼儿喂养习惯IYCF Practices 31
C2. 妈妈母乳喂养细节Breastfeeding Details 35
C3. 微量营养补充Micronutrient Supplements 37
D. 喂养知识与态度Feeding Knowledge and Attitude 38
D1. 母乳喂养自我效能Breastfeeding Efficacy 38
D2. 母乳准备自我效能 Efficacy in Preparation to Breastfeed 39
D3. *母乳喂养态度Breastfeeding Attitude 40
D4. 母乳喂养家庭支持 Breastfeeding Family Support 41
D5. 母乳喂养社会支持Advice About Breastfeeding 42
D6. *喂养知识Knowledge 44
D7. *喂养信息来源Feeding Information Sources....................45
E. 疾病与医疗卫生服务Disease & Health Services....................47
E1. 婴幼儿健康服务Newborn and Child Health Services....................47
E2. 疾病与医疗Illness and Medical Care....................47
E3. 影响婴幼儿健康的行为Behaviors Influencing Child Health 49
E4. 妈妈/孕妇保健Perinatal Care 50
E5. 妈妈/孕妇营养Perinatal Nutrition....................51
F. 怀孕史Pregnancy History 53
G. 心理健康Psychological Well-Being 53
G1. *抑郁、焦虑与压力 Depression, Anxiety, and Stress Scales (DASS-21) 53
G2. 孕产妇抑郁 perinatal depression....................54
H. *决策力Decision-Making....................55
I. *社会支持Perceived Social Support....................57
J. 育儿开销 Child Expenses....................58
K. 养育人社会网络调查表 The social network of the caregivers....................59
L. 体检结果 Physical Exam Results....................60

* 问卷单元由两位养育人分开回答Module to be answered by both caregivers separately.

指导语：

【如果宝宝已经出生】

您好！我们是四川大学的调研员，正在开展一项有关孕产妇、婴幼儿营养健康方面的调研。为了解您平时是怎么照顾宝宝的，我们会问您一些问题，希望您根据真实情况准确回答。同时，我们的专业护士会给您的宝宝测量身高、体重、贫血状况，并给宝宝的妈妈进行简单的体检，来评估宝宝和妈妈的身体健康状况。

【如果宝宝未出生】

您好！我们是四川大学的调研员，正在开展一项有关孕产妇、婴幼儿营养健康方面的调研。为了解孕妇的营养健康状况，我们会问您一些问题，希望您根据真实情况准确回答。同时，我们的专业护士会给正在孕期的您测量身高、体重、贫血状况，来评估您的身体健康状况。

您的参与是完全自愿的。答题开始后，您也可以拒绝回答我的任何问题。我们将对您的个人信息保密。

您同意参加我们的项目吗？

1=同意

2=不同意，请说明原因 ________

表头 Header

调查日期Date of investigation
调查员编码 Investigator ID
调查员姓名Name of investigator
县County
镇Township
村Village
组/自然村 Group/Natural Village
家庭编码Family code
第一养育人的姓名Mother's name
户主姓名Household Head Name

A. 家庭成员基本信息Household Roster

下面我想了解一下你家主要家庭成员的信息，包括宝宝的第一养育人（孕妇算作第一养育人）、第二养育人、父母、兄弟姐妹以及最近一年所有在家居住三个月以上的家庭成员

I'd like to get some information about the main members of your household, including the index child's primay caregiver, secondary caregiver, parents, siblings, & any family member that resided in the household for at least three months in the past year

编号 Member ID	与宝宝关系【不用读选项】Relationship with baby [do not read the options]	年龄（周岁）Age	教育程度【不用读选项】Highest education [do not read the options]	主要工作【不用读选项】Primary Occupation [do not read the options]	总体健康状况如何？overall health status of this family member?	是宝宝的第几养育人？Which caregiver?	宝宝第一/二养育人的姓名 name of primary/ secondary caregiver	宝宝第一/二养育人的联系方式（如果没有，写999）contact of primary/ secondary caregiver (if none, 999)	宝宝第一/二养育人的微信（如果没有，写999）Wechat ID of primary/ secondary caregiver (if none, 999)	宝宝第一/二养育人的QQ（如果没有，写999）QQ ID of primary/ secondary caregiver (if none, 999)
**1**	**2**	**3**	**4**	**5**	**6**	**7**	**8**	**9**	**10**	**11**
	1=父亲 Father<br>2=母亲 Mother<br>3=祖父 Paternal grandfather<br>4=祖母 Paternal grandmother<br>5=外祖父 Maternal grandfather<br>6=外祖母 Maternal grandmother<br>7=亲哥哥 Brother<br>8=亲姐姐 Sister	岁 Year	1=小学未毕业及以下did not graduate from primary school<br>2=小学毕业 graduated from primary school<br>3=初中毕业lower middle school degree<br>4=高中毕业upper middle school degree	1=学龄前 younger than schooling age<br>2=学生 student<br>3=务农 farming<br>4=打工 working for others<br>5=自营工商业 self-employed business<br>6=国家工作人员 government worker	1=非常差very poor<br>2=较差poor<br>3=一般fair<br>4=较好good<br>5=非常好 excellent	1=第一养育人primary caregiver<br>2=第二养育人secondary caregiver<br>3=都不是 neither => 下一人(skip to the next person)				

	9=其他【请说明】Other (please specify)		5=中等技术学校、职业学校毕业 technical or vocational degree<br>6=大专或大学毕业 university or college degree<br>7=硕士及以上 master’s degree or higher<br>999=不知道don’t know	7=在家带小孩 caregiver for children<br>8=无工作not working<br>9=其它（请说明）) other (please specify) ______						
01	第一养育人<br>Primary Caregiver									
02	第二养育人<br>Secondary Caregiver									
03										
04										
05										
06										
07										

B. 家庭背景 Family background

B1. 家庭信息Household Info

1.	宝宝出生的年月日或预产期 (阳历；举例，2019年1月1日应写成20190101）？<br>The child's (expected) birth date (western calendar)?	____年year ____月month ____日day
2.	宝宝妈妈的婚姻状况？Mother's current marital status?	1=已婚married<br>2=离婚divorced<br>3=丧偶widowed<br>4=未婚unmarried<br>5=其它，请说明other, specify______
3.	宝宝的妈妈有亲哥哥吗？Does the child's mother have an older brother?	1=是Yes<br>2=否No
4.	宝宝的爸爸有亲哥哥吗？Does the child's father have an older brother?	1=是Yes<br>2=否No
5.	宝宝是否出生? Has the child been born yet?	1=是Yes<br>2=否No→Skip to 14跳到第14题
6.	宝宝的性别? child's sex?	1=男boy<br>2=女girl
7.	宝宝的名字？child's name?	
8.	宝宝的妈妈现在是否和宝宝住在一起？Does the mother live with the child?	1=是Yes→Skip to 11跳到第11题<br>2=否No
9.	宝宝的妈妈为什么没有住在一起？？Why did the mother leave?	1=离家外出打工Migrated for work<br>2=其他，请注明Other reasons (please specify)
10.	宝宝的妈妈在宝宝几个月大的时候离开？How old was the child when the mother left?	____月month
11.	宝宝的爸爸现在是否和宝宝住在一起？Does the father live with the child?	1=是Yes→ Skip to 14跳到第14题<br>2=否No
12.	宝宝的爸爸为什么离开？Why did the father leave?	1=离家外出打工Migrated for work<br>2=其他，请注明Other reasons (please specify)
13.	宝宝的爸爸在宝宝几个月大的时候离开？How old was the child when the father left?	____月month
14.	去年一整年您家总收入大概有多钱？What was your family's total income last year?	____ 万元in 10 thousand RMB
15.	您家里有自来水吗？ Do you have tap water at home?	1=有 Yes<br>2=没有 No
16.	您家里有热水器（包括太阳能）吗？Do you have a water heater at home (incl. solar heater)?	
17.	您家里有洗衣机吗？Do you have a washing machine at home?	
18.	您家里有电脑吗？Do you have a computer at home?	
19.	您家里能够连上互联网吗，包括wi-fi或宽带？Do you have internet at home (including wi-fi，broadband)？	
20.	您家里有冰箱吗？Do you have a refrigerator at home?	

21. 您家里有空调吗？Do you have an air conditioner at home?	
22. 您家里有摩托车/电动车吗？Do you have a motorbike/electric bike?	
23. 您家里有小轿车/货车吗？Do you have a car?	
24. 您家冬天主要是怎么取暖的？What do you use for heating in the winter?	1=火盆brazier<br>2=有烟囱的炉子stove with chimney/pipe<br>3=没有烟囱的炉子 stove without chimney/pipe<br>4=电取暖器 electric heater<br>5=空调 air conditioner<br>6=其它，请注明other, please specify____
25. 你家的厕所是什么类型的？（如果有多种厕所，填最好的类型）What kind of toilet facilities does your household have?	1=没有no bathroom<br>2=冲水马桶 flush toilet<br>3=水泥坑 cement open pit<br>4=土坑earth open pit<br>5 =其它（请注明______）other (specify: _________)
26. 您家做饭主要用什么燃料？What type of fuel does your household mainly use for cooking?	1=煤coal<br>2=电electricity<br>3=煤油kerosene<br>4=液化气LPG<br>5=天然气natural gas<br>6=木柴、柴草等wood, sticks/straw,etc.<br>7=木炭charcoal<br>8=其它 other (请注明 specify: ___________)
27. 【观察题】我们想看看您家做饭的地方。您能带我看看您家人是在哪里做饭的吗？[Observation] We would like to have a look at the places that your household uses to cook food. Can you please show me where members of your household cook?	1=观察了，有独立厨房 observed, separate kitchen<br>2=观察了，非独立厨房 observed, no separate kitchen<br>3=没有观察 not observed
28. 【观察题】我们想看看您家洗手的地方。您能带我看看您家人是在哪里洗手的吗？[Observation] We would like to learn about the places that your household uses to wash their hands. Can you please show me where members of your household most often wash their hands?	1=观察了，固定地点 observed, fixed place<br>2=观察了，非固定地点 observed, mobile<br>3=没有观察not observed → 结束本单元end of section
29. 【观察题】【观察洗手处是否有肥皂、洗涤剂等其它清洁用品。记录观察结果。】[Observe presence of soap, detergent, or other cleansing agent at the place for handwashing. Record observation.]	1=有肥皂，香皂或洗涤剂（肥皂块、洗手液、洗涤精等）soap or detergent (bar soap, liquid soap, detergent, etc.)<br>2=没有none

B2. 妈妈外出情况Maternal Migration

目标样本：孕妇或0-6个月宝宝的母亲<br>ELIGIBILITY CHECK: Is the respondent a pregnant woman or mother of children 0 – 6 months of age?<br>1=yes 2=no → skip this section	
问题 Questions	选项 Options
1. 您是从哪里嫁过来的？From where did you marry into this family?	1=本乡镇from this village<br>2=非本乡镇not from this vilalge
2. 您之前外出工作过吗Have you migrated for work before?	1=有Yes<br>2=没有No → skip to 6
3. 您上一份外出工作是在哪里Where did you work for your last migrant job?【不要念选项do not read choices】	1=本乡镇 within the town<br>2= 本县非本镇in the same county but another town<br>3= 本市非本县in the same prefecture but another county<br>4=本省其他市in the same province but another prefecture<br>5=其他省 in another province<br>6=其他请说明other
4. 您上一份外出工作的平均月收入是多少How much was your average monthly income in your last migrant job?	______人民币RMB
5. 您最近一次打工结束、回到村子里是什么时候？When did you move to the village from your last migrant job?	__年 year ___月month
6. 您接下来计划在孩子多大时离家外出工作At what age of your child do you plan to migrate for work?	1=______月months → 跳至第8题skip to Q8<br>2=没有外出工作的计划 no plan to migrate for work<br>3=目前不确定 not sure→ 跳至第8题skip to Q8
7. 没有离家外出工作计划的原因是什么Why are you not planning to migrate for work?【不要读选项 do not read the choices】	1=想亲自带孩子I want to raise the child on my own<br>2=没人帮忙带孩子 no one to take care of your children except yourself<br>3=不放心其他人带孩子 worried about others taking care of your child<br>4=家里面有活走不开can't pull away from household duties<br>5=没有工作的打算no plan to work<br>6=其他，请注明other______<br>→结束本部分→ end of section
8. 如果有离家外出工作的计划，是否会带孩子一起外出？If yes, would you bring your child with you?	1=是yes<br>2=否no<br>3=目前不确定not sure
9. 您打算离家外出去哪里工作Where do you plan to work?【不要念选项do not read choices】	1=本乡镇in the same town<br>2= 本县非本镇in the same county but another town<br>3= 本市非本县in the same prefecture but another county<br>4=本省其他市in the same province but another prefecture<br>5=其他省 in another province<br>6=目前不确定not sure for now<br>7=其他，请说明other, specify________

C. 喂养行为 Feeding Practices

C1. 婴幼儿喂养习惯IYCF Practices

- WHO indicators for assessing infant and young child feeding practices: http://apps.who.int/iris/bitstream/handle/10665/44306/9789241599290_eng.pdf;jsessionid=FEB01F6AAC8D70A5A98FBDB6B32CF939?sequence=1
    - Food groups based on CEEE previous survey "CEEE QingLing Cohort follow-up survey"

核实目标样本：该样本是0-6月龄宝宝吗？<br>ELIGIBILITY CHECK: Is the index child 0-6 months of age?<br>1=yes 2=no → skip this section	
1. 这个宝宝喝过母乳吗？Has the child ever been breastfed?	1=是yes<br>2=否no
2. 宝宝出生后多久第一次吮吸乳头？How soon after birth did the baby suckle at the breast for the first time?<br>[如果少于1小时，圈出"1"，并记录00小时；<br>如果少于24小时，圈出"1"并记录小时数，从01到23；<br>如果大于24小时，圈"2"并记录完成的天数。]<br>*[If respondent reports she put the infant to the breast in less than 1 hour, circle '1' for hours AND RECORD '00' hours. If less than 24 hours, circle '1' and record number of completed hours, from 01 to 23. Otherwise, circle '2' and record number of completed days.]*	1=小时hour |__|__| 2=天days |__|__| 3=从来没有吮吸过乳头never<br>999=不知道don't know
3. What was the child fed first after birth?<br>宝宝出生后第一口喂的是什么？【不要读选项】<br>DO NOT READ RESPONSE OPTIONS	1=母乳/初乳breastmilk/colostrum<br>2=配方奶formula<br>3=水water<br>4=其它（注明）other (specify)______<br>999=不知道don't know
4. 是否给宝宝喂了初乳（初乳是产后一周内产生的淡黄色、粘稠的母乳）Was the child fed colostrum?	1=是yes<br>2=否no<br>999=不知道don't know
5. 宝宝在医院的时候被喂过水吗？Was the baby fed water at any time in the hospital?	1=是yes<br>2=否no<br>999=不知道don't know
6. 宝宝在医院的时候被喂过奶粉吗？Was the baby fed formula at any time in the hospital?	1=是yes<br>2=否no<br>999=不知道don't know
7. 宝宝在医院的时候被喂过糖水吗？Was the baby fed sugared water at any time in the hospital?	1=是yes<br>2=否no<br>999=不知道don't know
8. Was the child breastfed yesterday during the day or at night?<br>昨天（包括白天和晚上）宝宝是否喝过母乳？	1=是yes<br>2=否no → 第10题 skip to Q10<br>999=不知道don't know→ 第10题 skip to Q10
9. 宝宝昨天（包括白天和晚上）喝了几次母乳？How many times yesterday during the day or at night was the child breastfed?	___次times<br>999=不知道don't know
10. 宝宝昨天（包括白天和晚上）有没有喝水？Did the child have any plain water yesterday?	1=是yes<br>2=否no<br>999=不知道don't know
11. Did the child have any infant formula yesterday?<br>宝宝昨天（包括白天和晚上）有没有喝婴儿配方奶粉？	1=是yes<br>2=否no → 第14题 skip to 14

	999=不知道don’t know→ 第14题 skip to 14
12. 宝宝昨天（包括白天和晚上）喝了几次配方奶粉？How many times yesterday did the child consume infant formula?	___次times<br>999=不知道don’t know
13. 宝宝昨天（包括白天和晚上）喝了多少毫升的配方奶粉？How many milliliters of infant formula did the child consume yesterday?	___毫升ML<br>999=不知道don’t know
14. 什么时候开始给宝宝喂配方奶粉? At what age did the baby start to have formula?	______月month<br>0=从出生就开始喝配方奶 from birth<br>888=未添加奶粉has not started
15. 您多久洗一次宝宝的奶瓶？How often do you wash your baby’s bottles?【不要读选项do not read the choices】	0=没有/不用奶瓶Don’t have/don’t use bottles → 跳到17题skip to Q17<br>1=每次用完就洗after every use<br>2=每天Every day<br>3=每周Every week<br>4=每两周Every two weeks<br>5=每个月Every month<br>6=少于每个月一次Less than once a month<br>7=从来不洗Never →跳到17题 skip to Q17
16. 通常，您怎么给宝宝洗奶瓶How do you wash your baby’s bottles?【不要读选项do not read the choices】	1=只用冷水冲洗Rinse with cold water<br>2=用开水煮或者烫boil or rinse with boiling water<br>3=用特制洗奶瓶器Use a designated bottle-sanitizing device<br>4=用洗洁精和水洗Wash with soap and water<br>6=其它，请注明Other, specify:_______
17. Did the child have any milk such as tinned, powdered, or fresh animal milk yesterday?宝宝昨天（包括白天和晚上）有没有喝新鲜动物奶（比如牛奶、羊奶）或动物奶粉？	1=是yes<br>2=否no → skip to 19<br>999=不知道don’t know→ skip to 19
18. 宝宝昨天（包括白天和晚上）喝了几次新鲜动物奶或动物奶粉？ How many times yesterday did the child consume any milk of the kinds just asked about?	___次times<br>999=不知道don’t know
19. 宝宝昨天（包括白天和晚上）有没有喝过果汁或果汁饮料？Did the child have any juice or juice drink yesterday?	1=是yes<br>2=否no<br>999=不知道don’t know
20. 宝宝昨天（包括白天和晚上）有没有喝过汤？Did the child have any clear broth yesterday?	1=是yes<br>2=否no<br>999=不知道don’t know
21. 宝宝昨天（包括白天和晚上）有没有喝酸奶？Did the child have any yogurt yesterday?	1=是yes<br>2=否no → skip to Q23<br>999=不知道don’t know → skip to Q23
22. 宝宝昨天（包括白天和晚上）有没有喝粥？Did the child have any thin porridge yesterday?	1=是yes<br>2=否no<br>999=不知道don’t know

23. 宝宝昨天（包括白天和晚上）有没有喝益生菌乳品饮料，比如养乐多？Did the child have any probiotic dairy drink such as Yakult yesterday?	1=是yes<br>2=否no<br>999=不知道don't know
24. 宝宝昨天（包括白天和晚上）有没有喝其他饮品，比如汽水、茶、豆浆等Did the child have any other liquid yesterday (e.g., sugar water, soda, tea, soy milk)?	1=是yes<br>2=否no<br>999=不知道don't know
25. 宝宝是在几月龄开始添加辅食（除了母乳和配方奶之外给宝宝提供主要能量来源的液体、半固体、固体食物）？ At what age did the baby start to have complementary foods (liquid, semi-solid, or solid foods other than breastmilk or formula that provide nutrients)?	______月month<br>888=还未添加辅食has not started yet→Q43<br>999=不知道don't know
26. 宝宝昨天是否吃了米汤、粥、面汤、馒头或米饭等主食类食物？Did the baby eat any staple food yesterday, such as rice porridge, flour porridge, steamed bun or rice	1=是yes<br>2=否no<br>999=不知道don't know
27. Did the baby eat any yellow or orange food yesterday, such as pumpkin, carrot or red sweet potato宝宝昨天是否吃了南瓜、胡萝卜、红心红薯等里面是黄色或橙色的食物	
28. 宝宝昨天是否吃了土豆、山药、白萝 卜、白心红薯等根茎类食物？Did the baby eat any root and stem vegetables yesterday, such as potato, yam, radish, white sweet potato	
29. Did the baby eat any leafy dark green vegetables yesterday? 宝宝昨天是否吃了深绿色叶子菜？（举例：菠菜、豌豆尖）	
30. Did the baby eat any red or yellow fruits such as persimmon, apricot, watermelon, cantaloupe or tomato yesterday? 宝宝昨天是否吃了红色或黄色的水果，如柿子、杏、西瓜、哈密瓜或番茄?	
31. Did the baby eat any other fruits or vegetables yesterday? 宝宝昨天是否吃了其他水果或蔬菜?	
32. Did the baby eat any organ meats such as animal liver, kidney or heart yesterday? 宝宝昨天是否吃了内脏类食物，如动物肝、肾或心脏	
33. Did the baby eat any other meat or meat products (e.g., chicken, duck, pork, beef, lamb, etc.) yesterday? 宝宝昨天是否吃了肉类或肉类产品（如鸡肉、鸭肉、猪肉、牛肉、羊肉等）	
34. Did the baby eat any eggs yesterday? 宝宝昨天是否吃了鸡蛋	
35. Did the baby eat any fresh or dried fish, shellfish or seafood yesterday? 宝宝昨天是否吃了鱼类、贝类或海鲜类食物?	
36. Did the baby eat any beans, peas, lentils, nuts or seeds yesterday? Please note: Drinking soy milk counts 宝宝昨天是否吃了扁豆等豆类、豆制品或坚果、种子? 注意：喝过豆浆算吃过豆制品	
37. Did the baby eat any dairy products such as cheese and yoghurt yesterday?宝宝昨天是否吃了乳制品，如奶酪和酸奶	
38. Did the baby eat any oil (including cooking oil) and meat fat yesterday?宝宝昨天是否吃了油（包括炒菜用的油）、肥肉（含肉汤）等脂肪类的食物	
39. Did the baby eat any snacks such as biscuit, dessert, candy, chocolate and cake yesterday? 宝宝昨天是否吃了零食，如饼干，甜点，糖果，巧克力和蛋糕	
40. Did the baby eat any condiments for flavor, such as salt, chilies, spices, cilantro, ginger, garlic, or fish powder yesterday?宝宝昨天是否吃了调料，如盐、辣椒、香料、香菜、姜、蒜或味精?	
41. 该宝宝昨天是否吃过任何强化铁的婴儿食品，比如强化铁米粉、面条？Yesterday, during the day or night, did the child	

consume any iron-fortified foods designed specifically for infants and young children, e.g., fortified baby cereal or noodles?	
42. Yesterday, how many times did the child have solid food (such as rice and meat buns), semi-solid food (something thick, such as porridge) or soft food (such as flour puree, fruit puree and vegetable puree)? 昨天，宝宝吃了多少次固体食物（如米饭和肉包子）、半固体食物（稠的，如粥）或软食物（如面糊、果泥和蔬菜泥）？	_____次Times<br>999=不知道don't know
43. 您一般在什么情况下喂宝宝？【不要读选项;可多选】？When do you decide that it is time to feed your baby [do not read list; multiple choice]?	1=到时间了（大人要吃饭了或者到了饭点）it is time to eat (family meal time or clock time)<br>2=到宝宝该饿了的时候it is the time the baby is usually hungry<br>3=当宝宝用手势或语言要求吃的时候（还没有闹的时候）when the baby asks with gestures or words, but is not upset<br>4=当宝宝哭或捣乱的时候when the baby cries or fusses<br>5=其它（请注明）other________
44. 当宝宝不愿意吃的时候您会怎么做？【多选；不要读选项】What do you do when a child refuses to eat?<br>[mark all that are mentioned and DO NOT read list]	1=坚持要喂完insist the child eats the food<br>2=混入宝宝喜欢吃的食物mix in another food the child likes<br>3=用语言鼓励、哄宝宝provide verbal encouragement<br>4=给宝宝奖励offer a reward<br>5=暂停喂、等一会儿再试stop and try later<br>6=停止喂饭、到下一餐前不给宝宝吃任何东西stop and offer no food until next usual meal<br>7=惩罚或者吓唬宝宝punish or threaten to punish the child in some way<br>8=喂配方奶或配方奶粉feed the baby formula<br>9=其它（请注明）<br>other______________

C2. 妈妈母乳喂养细节Breastfeeding Details

CHECK FOR ELIGIBILITY: 核实是否是目标样本<br>Is the respondent mother of a child 0-6 months of age? 该样本是0-6月龄宝宝的妈妈吗？<br>1. Yes是<br>2. No → skip to next section否→跳到下一部分

1. 您曾经给宝宝喂过、或者<u>尝试</u>喂过母乳吗？Did you ever breastfeed or try to breastfeed the baby?	1=是yes → skip to Q3<br>2=否no
2. 您决定不给宝宝喂母乳的原因有哪些【可多选；不要读选项】？ What are the reasons for your decision not to breastfeed your baby? [Do not read the options.]<br><br>→ 结束本单元end this section	1= 宝宝生病 baby was sick<br>2= 没有奶not enough breastmilk<br>3= 医生建议不要母乳喂养doctor suggested not to breastfeed.<br>4=宝宝妈妈生病mom was sick.<br>5= 奶粉和母乳一样好或者比母乳更好formula is as good as breastfeeding or formula is better.<br>6=太麻烦too inconvenient.<br>7=以前喂过母乳但是不喜欢或者不顺利 tried before and didn't like it or it didn't work out.<br>8=我想有自己的时间（比如，离开家几个小时也不用担心孩子要喂奶）I wanted to be able to leave the baby for several hours at a time.<br>9=家务太多too many household duties.<br>10=我计划恢复工作I planned to go back to work.<br>11=我想自己掌控自己的身体，不必因为喂奶改变自己的生活方式。I wanted my body back to myself.<br>12=别人想喂宝宝Someone else wanted to feed the baby.<br>13=宝宝的父亲不希望我喂母乳。The baby's father didn't want me to breastfeed.<br>14=宝宝的（外）祖母不希望我喂母乳。The baby's grandmother didn't want me to breastfeed.<br>15=我想用的避孕方式不能进行母乳喂养I wanted to use contraception that can't be used while breastfeeding.<br>16=其它，请注明other: specify____

宝宝的妈妈在母乳喂养的<u>**最初两周**</u>遇到过以下问题吗？【逐一问Q3-21到中列出的问题】did the baby's mom have any of the following concerns or difficulties in the <u>first 2 weeks</u> of breastfeeding? Ask Q3 – 21 one by one.

3. 乳房疼痛Breast pains	1=有yes<br>2=没有no<br>999=不清楚don't know
4. 背部疼痛 back pains	
5. 孩子吮吸得不好baby had trouble sucking or latching on	
6. 乳头酸疼、破裂或流血sore, cracked, or bleeding nipples	
7. 母乳不足not enough breastmilk	
8. 剖宫（腹）产影响母乳喂养C-Section affected breastfeeding	
9. 阴道侧切影响母乳喂养episiotomy (cut vagina)	
10. 医生建议不要母乳喂养doctor suggested not to breastfeed	
11. 宝宝呛奶。baby chocked when breastfeeding.	

12. 宝宝不能按时醒来喝奶。baby wouldn’t wake up to nurse regularly enough.	
13. 宝宝对吃奶不感兴趣或走神。baby was not interested in nursing or got distracted.	
14. 宝宝吃奶太频繁。Baby nursed too often.	
15. 下奶太慢。It took too long for breast milk to come in.	
16. 宝宝长得不够快或者体重太轻。baby didn’t gain enough weight or lost too much weight	
17. 没有充足时间给孩子喂奶not enough time to feed child	
18. 乳房感染（例如脓肿、真菌感染等）Infection of the breasts (e.g., abscess, yeast)	
19. 奶管堵塞clogged milk duct	
20. 乳房肿胀/涨奶breast engorgement	
21. 漏奶太多breasts leaked too much.	
22. 您给宝宝喂过除了母乳以外的食物吗，例如奶粉、动物奶、果汁、汤、粥、茶、汽水等？Have you introduced any foods other than breastmilk to your child, such as formula, animal milk, juice, broth, yogurt, porridge, tea, soda, etc.?	1=是yes<br>2=否no → end of section

23. 您为什么给宝宝喂除了母乳之外的食物？【多选；不要读选项】Why did you start introducing foods other than breastmilk? [multiple choice; do not read answer]

Child Reasons宝宝的原因

1. 宝宝生病 baby was sick
2. 宝宝呛奶。baby choked.
3. 宝宝不能按时醒来喝奶。baby wouldn’t wake up to nurse regularly enough.
4. 宝宝对吃奶不感兴趣或走神。baby was not interested in nursing or got distracted.
5. 宝宝吃奶太频繁。Baby nursed too often.
6. 宝宝长得不够快或者体重太轻。baby didn’t gain enough weight or lost too much weight
7. 宝宝吮吸得不好baby had trouble sucking or latching on
8. 宝宝需要其它食物（包括水）。Baby needs other foods (including water).

Breastfeeding problems 母乳喂养问题

9. 母乳不足not enough breastmilk
10. 乳房疼痛Breast pains
11. 背部疼痛 back pains
12. 乳头酸疼、破裂或流血sore, cracked, or bleeding nipples
13. 奶管堵塞clogged milk duct
14. 乳房肿胀/涨奶breast engorgement
15. 乳房感染（例如脓肿、真菌感染等）Infection of the breasts (e.g., abscess, yeast)
16. 下奶太慢。It took too long for breast milk to come in.
17. 漏奶太多breasts leaked too much.

Breastfeeding attitudes or preference母乳喂养态度和偏好

18. 奶粉和母乳一样好或者比母乳更好formula is as good as breastfeeding or formula is better.
19. 太麻烦too inconvenient.
20. 以前喂过母乳但是不喜欢或者不顺利。 tried before and didn’t like it or it didn’t work out.
21. 我想自己掌控自己的身体，不必因为喂母乳改变自己的生活方式。I wanted my body back to myself.

Benefits of other foods 其它食物的的好处

22. 添加其它食物后宝宝体重增加得更快Better weight gain when I introduced other foods.
23. 添加其它食物后宝宝哭得更少Baby cries less when I introduced other foods.
24. 添加其它食物后宝宝睡得更好Baby sleeps better when I introduced other foods.

25. 添加其它食物后妈妈睡得更好Mom sleeps better when I introduced other foods.<br>26. 宝宝更喜欢其它食物The baby likes it better when I introduced other foods.<br><br>Maternal reasons 妈妈的原因<br>27. 宝宝妈妈生病mom was sick.<br>28. 医生建议不要母乳喂养doctor suggested not to breastfeed<br>29. 剖宫（腹）产影响母乳喂养C-Section affected breastfeeding<br>30. 阴道侧切影响母乳喂养episiotomy (cut vagina)<br>31. 想一次能离开孩子几个小时I wanted to be able to leave the baby for several hours at a time.<br>32. 家务太多too many household duties.<br>33. 计划回去工作。I planned to go back to work.<br>34. 想用母乳喂养时不能用的避孕方式I wanted to use contraception that can't be used while breastfeeding.<br>35. 没有充足时间母乳喂养孩子not enough time to feed child<br><br>Other family members 其它家庭成员<br>36. 别人想喂宝宝。Someone else wanted to feed the baby.<br>37. 宝宝的爸爸不希望我喂母乳。The baby's father didn't want me to breastfeed.<br>38. 宝宝的奶奶或外婆不希望我喂母乳。The baby's grandmother didn't want me to breastfeed.<br>39. 其它原因，请注明 other reason, specify__________

C3. 微量营养补充Micronutrient Supplements

CHECK FOR ELIGIBILITY:<br>Is the index child 0-6 months of age? 该样本是0-6月龄宝宝吗？<br>1. Yes是<br>2. No → skip to next section否→跳到下一部分	
1. 宝宝吃过含铁的微量元素补充剂吗（例如粉末、滴剂等）Has the child ever consumed any micronutrient supplement with iron (e.g., powder, drops)?	1=是yes<br>2=否no → 跳至skip to 5<br>999=不知道don't know跳至skip to 5
2. 宝宝几个月大时开始吃含铁的微量元素补充剂？At what age did the child start consuming micronutrient supplement with iron?	___月months<br>999=不知道don't know
3. 宝宝几个月大时停止吃含铁的微量元素补充剂？At what age did the child stop consuming micronutrient supplement with iron?	____月months<br>888 还在吃still consuming<br>999=不知道don't know
4. 该宝宝昨天是否吃了含铁的微量元素补充剂？Yesterday, during the day or night, did the child consume any iron supplement?	1=是yes<br>2=否no<br>999=不知道don't know
5. 宝宝出生后您是否收到过免费营养包？ Have you received free micronutrient supplement packages (YYB) since the baby's birth?	1=是yes<br>2=否no → 结束本单元end of section
6. 你给自己的宝宝喂过免费的营养包吗？Have you fed your child these free YYB?	1=是yes<br>2=否，跳题no → 结束本单元end of section
7. 上一周你给宝宝喂了多少袋营养包？ How many packages of YYB did you feed your child last week?	____ 袋packages

D. 喂养知识与态度Feeding Knowledge and Attitude

D1. 母乳喂养自我效能Breastfeeding Efficacy

Breastfeeding Self-Efficacy Scale – Short Form

- Original scale: Dennis C. The Breastfeeding Self-Efficacy Scale: psychometric assessment of the short form. J Obstet Gynecol Neonatal Nurs. 2003;32(6):734-744.
- Validated in Chinese: Ip, Wan-Yim, et al. "The short form of the breastfeeding self-efficacy scale as a prognostic factor of exclusive breastfeeding among mandarin-speaking Chinese mothers." Journal of Human Lactation 32.4 (2016): 711-720.
    - The validated scale (Guangzhou) only included the first 13 questions.

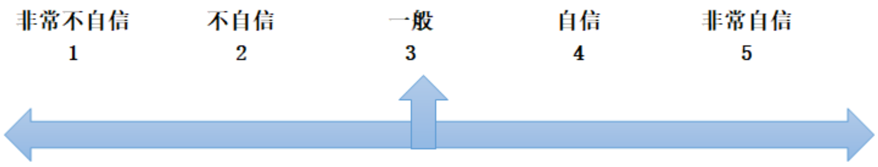

目标样本：该样本是正在喂母乳的妈妈吗？ Is the respondent a breastfeeding mom?<br>1=是 yes 2=否（跳过此部分）no → skip to next section	
你对下面的陈述有多自信？How confident are you in the following statement?<br>1=非常不自信not at all confident<br>2=不自信 not confident<br>3=一般 okay<br>4=自信 confident<br>5=非常自信always confident	答案<br>Answer
1. 我总能确保宝宝得到充足的母乳Determine that my child is getting enough milk	
2. 我总能做好母乳喂养，就像以前我总能很好地应付其他具有挑战性的事一样 Successfully cope with breastfeeding like I have with other challenging tasks	
3. 我总能只给宝宝母乳喂养而不添加配方奶Breastfeed my child without using formula as a supplement	
4. 我总能确保宝宝在整个吃奶过程中能够正确地含住乳头吮吸Ensure that my child is properly latched on for the whole feeding	
5. 我总能将母乳喂养的状况控制到令我满意的程度Manage the breastfeeding situation to my satisfaction	
6. 即使在孩子哭的时候，我也总能将哺乳进行下去Manage to breastfeed even if my child is crying	
7. 我总能保持想要母乳喂养的愿望Keep wanting to breastfeed	
8. 喂奶时即便有家人在场，我也能心情放松而不会感到尴尬Comfortably breastfeed with my family members present	
9. 我总能对自己母乳喂养的状况很满意Be satisfied with my breastfeeding experience	
10. 虽然母乳喂养比较耗时，但我也能应付Deal with the fact that breastfeeding can be time-consuming	
11. 我总能只用一侧乳房就能把孩子喂饱Finish feeding my child on one breast before switching to the other breast	
12. 每次喂奶我都能一气呵成而不会间断地完成Continue to breastfeed my child for every feeding	
13. 我总能满足孩子对母乳的需求量Manage to keep up with my child’s breastfeeding demands	
14. 在宝宝不到六个月时，我总能只给宝宝喂母乳，不喂一滴水I can always exclusively breastfeed without my child receiving even a drop of water	
15. 在宝宝不到六个月时，我总能阻止别人给我的宝宝喂母乳以外的食物，例如婴儿配方奶粉，牛奶，粥，果汁，茶等[不论给什么]I can always stop someone from	

	trying to feed my child liquids or foods other than breast milk (e.g. infant formula, milk, porridge, juice, tea [whatever is given]), before 6 months of age	
16.	我总是能够判断孩子是否吃饱了Tell when my child is finished breastfeeding	

D2. 母乳准备自我效能 Efficacy in Preparation to Breastfeed

Questions adapted from the Prenatal Rating of Efficacy in Preparation to Breastfeed Scale

McKinley EM, Knol LL, Turner LW, et al. The Prenatal Rating of Efficacy in Preparation to Breastfeed Scale: A New Measurement Instrument for Prenatal Breastfeeding Self-efficacy. *J Hum Lact*. 2018:089033441879904. doi:10.1177/0890334418799047

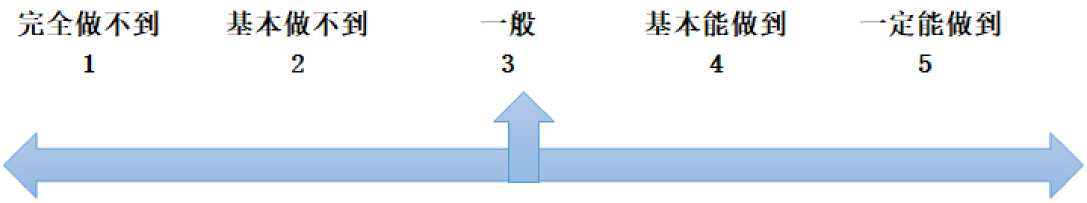

核实目标样本：该样本是孕妇吗？ Is the respondent a pregnant woman?<br>1=是 yes. 2=否（跳过此部分）no → skip to next section		
注意：用箭头给家长解释选项，并让家长看着箭头回答<br>(Use the above graph to explain this questions)		
1.	根据您现在的生活，您觉得您能克服母乳喂养带来的焦虑吗？ Overcome any anxiety you may feel about breastfeeding?	请在1到5之间选出一个数字，1代表完全做不到，5代表非常确定可以做到。Please provide a rating between 1 (can’t do) and 5 (highly certain can do)<br><br>1=完全不能cannot do<br>2=基本不能<br>3=一般<br>4=基本能<br>5=一定能highly certain can do
2.	根据您现在的生活，您觉得您能安排出时间给宝宝喂母乳吗？ Manage your time so you can breastfeed?	
3.	根据您现在的生活，您觉得您能应对母乳喂养带来的挑战吗？ Manage the possible challenges that may come with breastfeeding?	
4.	根据您现在的生活，您觉得您能想象到自己顺利给宝宝喂母乳的画面吗？ Visualize yourself being successful at breastfeeding?	
5.	根据您现在的生活，您觉得您能与其他妈妈或者孕妇讨论母乳喂养吗？ Discuss breastfeeding with other mothers or pregnant women?	
6.	根据您现在的生活，您觉得您能向其他正在喂母乳的妈妈问相关问题吗？ Ask another breastfeeding mother questions about breastfeeding?	
7.	根据您现在的生活，您觉得您能和您的好朋友讨论母乳喂养吗？ Talk about breastfeeding with your close friends?	
8.	根据您现在的生活，您觉得您能和您的家人讨论母乳喂养吗？ Talk about breastfeeding with family members?	
9.	根据您现在的生活，您觉得您能向别人解释母乳喂养的好处吗？ Explain the benefits of breastfeeding to another person?	
10.	根据您现在的生活，您觉得您能告诉别人如何喂母乳吗？ Explain how to breastfeed a child to another person?	
11.	根据您现在的生活，在遇到母乳喂养的问题时，您觉得您能找到解决方法吗？ Find the answers to your questions about breastfeeding?	
12.	根据您现在的生活，您觉得您能应对不支持母乳喂养的家人吗？ Handle family that do not support breastfeeding?	
13.	根据您现在的生活，您觉得您能应对不支持母乳喂养的朋友吗？ Handle friends that do not support breastfeeding?	

D3. *母乳喂养态度Breastfeeding Attitude

- Iowa Infant Feeding Attitude Scale
- Original: https://onlinelibrary.wiley.com/doi/pdf/10.1111/j.1559-1816.1999.tb00115.x
- Validated in China
  - Hong-Xia Dai, Xiang-Dong Guan, Xiao-Mao Li, Li-Ming You & Ying Lau (2013) Psychometric properties of a mainland Chinese version of the Iowa Infant Feeding Attitude Scale among postpartum women in China, Contemporary Nurse, 44:1, 11-20, DOI: 10.5172/conu.2013.44.1.11
  - Suggest to delete three questions (6, 10, 17). We can keep them for now.

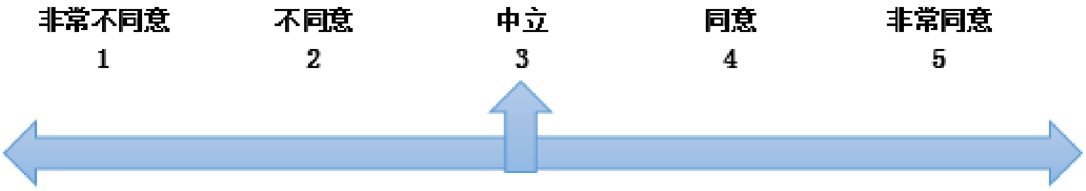

<table>
<tr><td colspan="2">Iowa Infant Feeding Attitude Scale
For each of the following statements, please tell me how much you agree or disagree by choosing the number that most closely corresponds to your opinion (1 = strong disagreement [SD], 2 = disagreement [D], 3 = neutral [N], 4 = agreement [A], 5 = strong agreement [SA]. You may choose any number from 1 to 5

对于下面的每一个陈述，请告诉我你同意或不同意的程度，选择与你的意见最接近的答案。1=非常不同意[SD]，2=不同意[D]，3=中立N [N]，4=同意A[A]，5=非常同意[SA]。你可以从1到5选择任何数字。
(注意：用箭头给家长解释选项，并让家长看着箭头回答）</td></tr>
<tr><td>1. 母乳喂养只在宝宝断奶前有好处The nutritional benefits of breastfeeding last only until the child is weaned from breast milk.</td><td rowspan="12">1=非常不同意 strong disagreement
2=不同意disagreement
3=中立neutral
4=同意agreement
5=非常同意 strong agreement</td></tr>
<tr><td>2. 配方奶粉喂养比母乳喂养更方便Formula-feeding is more convenient than breastfeeding.</td></tr>
<tr><td>3. 母乳喂养加强了妈妈和宝宝之间的联系Breastfeeding increases mother-infant bonding.</td></tr>
<tr><td>4. 母乳中缺乏铁Breast milk is lacking in iron.</td></tr>
<tr><td>5. 配方奶喂养比母乳喂养更易过量Formula fed babies are more likely to be overfed than breastfed babies.</td></tr>
<tr><td>6. 如果母亲打算外出打工，配方奶粉喂养是个更好的选择Formula feeding is the better choice if a mother plans to go back to work.</td></tr>
<tr><td>7. 喂配方奶会错过当妈妈的一大乐趣Mothers who formula feed miss one of the great joys of motherhood.</td></tr>
<tr><td>8. 妈妈不应该在公共场合喂奶，如餐馆Women should not breastfeed in public places such as restaurants.</td></tr>
<tr><td>9. 母乳喂养的宝宝比配方奶喂养的宝宝更健康Breastfed babies are healthier than formula fed babies</td></tr>
<tr><td>10. 母乳喂养比配方奶喂养更易过量Breastfed babies are more likely to be overfed than formula fed babies.</td></tr>
<tr><td>11. 如果妈妈进行母乳喂养，爸爸会感觉被冷落Fathers feel left out if a mother breast feeds.</td></tr>
<tr><td>12. 母乳是宝宝最理想的食物Breast milk is the ideal food for babies.</td></tr>
</table>

13. 母乳比配方奶更容易消化Breast milk is more easily digested than formula.	
14. 对宝宝来说，配方奶和母乳一样健康Formula is as healthy for an infant as breast milk.	
15. 母乳喂养比配方奶喂养更方便Breastfeeding is more convenient than formula.	
16. 母乳比配方奶便宜Breast milk is less expensive than formula.	
17. 偶尔喝酒的母亲不应该宝宝喂母乳A mother who occasionally drinks alcohol should not breastfeed her child.	
18. 配方奶喂养的宝宝更聪明。Formula makes the baby smarter.	

D4. 母乳喂养家庭支持 Breastfeeding Family Support

Zhu, Xiu, Luyan Liu, and Yan Wang. 2016. "Utilizing a Newly Designed Scale for Evaluating Family Support and Its Association with Exclusive Breastfeeding." Breastfeeding Medicine 11 (10): 526–31. https://doi.org/10.1089/bfm.2016.0090.

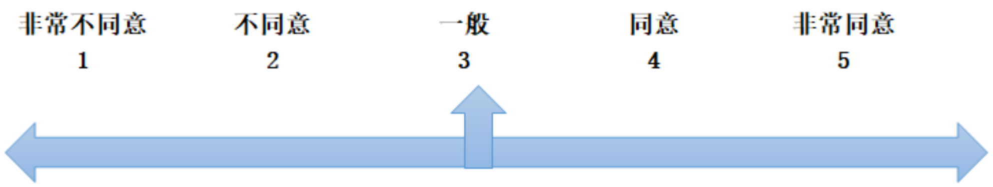

核实目标样本：该样本是0-6月龄宝宝的妈妈吗？<br>1 =是<br>2 =否→跳到下一部分<br>ELIGIBILITY CHECK: Is the respondent a mother of children 0 – 6 months of age?<br>1=yes<br>2=no → skip to next section	
您在多大程度上同意以下说法：To what extent do you agree with the following statement:	回答Response
1. 我的家人希望我纯母乳喂养我的宝宝。I think my family wants me to exclusively breastfeed my child.	1=非常不同意 strongly disagree<br>2=不同意 disagree<br>3=一般Neutral<br>4=同意 agree<br>5=非常同意 strongly agree
2. 我的家人认为我的奶水不足，需要给宝宝喂配方奶粉。My family believes that my lactation is inadequate and that I need to give formula to my child.	
3. 家人认为，充足的母乳来自持续的母乳喂养。My family thinks that abundant lactation comes from continuous breastfeeding.	
4. 我的家人认为母乳可以用配方奶粉代替。My family thinks that lactation can be replaced by formula.	
5. 我的家人将婴儿的哭声归因于没有好好地母乳喂养。My family attributes the child's crying to unsuccessful breastfeeding.	
6. 我在母乳喂养过程遇到困难时，家人经常鼓励我继续母乳喂养。In times of difficulty, my family encourages me to continue breastfeeding.	
7. 我的家人经常给我做各种下奶的饭菜。My family often prepares food that is good for lactation.	
8. 我的家人经常帮我照顾宝宝，以便让我好好休息。My family often helps me take care of the child to let me have a good rest.	
9. 如果母乳喂养期间有一些问题，我的家人经常会帮助我。If there are some problems during breastfeeding, my family often helps me out.	

D5. 母乳喂养社会支持Advice About Breastfeeding

检查是否是目标样本：该样本是孕妇或者0-6月龄宝宝的妈妈吗？<br>1 =是<br>2 =否→跳到下一部分<br>ELIGIBILITY CHECK:<br>Is the respondent a pregnant woman or mother of children 0 – 6 months of age?<br>1=yes<br>2=no → skip to next section	
1. 有人建议过你不要喂母乳吗？Were you ever discouraged from breastfeeding or told not to breastfeed?	1=是yes<br>2=否no → skip to Q4
2. 谁建议你不要喂母乳？【可多选】Who discouraged you or told you not to? (Multiple selections allowed)	1=村医 Village doctor<br>2=医院的医务人员Medical professional<br>3=宝宝的外婆Maternal grandmother<br>4=宝宝的奶奶Paternal grandmother<br>5=丈夫Husband<br>6=其他家庭成员，请注明：Other family member, specify: __________<br>7=朋友Friend<br>8=销售人员Salesperson<br>9=其他人，请注明Other, specify:____________
3. 你会听他的吗？（依据2生成二维表）<br>Would you listen to them?	1=会 Yes<br>2=不会 No
4. 别人建议您不要喂母乳的主要原因是什么？【不要读选项】What was the primary reason you were discouraged from breastfeeding? [do not read the choices]	1=剖腹产C-section<br>2=肝炎hepatitis<br>3=阴道侧切术episiotomy<br>4=避免黄疸to avoid jaundice<br>5=宝宝不会饿到、哭得少Baby will not be hungry and will cry less<br>6=妈妈奶水不足I did not have enough breastmilk<br>7=配方奶和母乳一样好、甚至比母乳更好Formula is just as good or better than breastmilk<br>8=喂母乳不方便It is inconvenient<br>9=宝宝体重增加不足Baby will not gain enough weight<br>10=宝宝体重增加太多Baby will gain too much weight<br>11=其它，请注明Other, specify_____
5. 有人鼓励过你喂母乳吗？Were you ever encouraged to breastfeed?	1=是yes<br>2=否no → skip to Q7
6. 谁曾鼓励过你喂母乳？【多选】Who encouraged you? (Multiple selections allowed)	1=村医 Village doctor<br>2=医院的医务人员Medical professional<br>3=宝宝的外婆Maternal grandmother<br>4=宝宝的奶奶Paternal grandmother<br>5=丈夫Husband<br>6=其他家庭成员，请注明：Other family member, specify: __________<br>7=朋友Friend<br>8=销售人员Salesperson

	9=其他人，请注明Other, specify:____________
7. 你会听他们的嘛？（依据6生成二维表）	1=会<br>2=不会
8. 这个人建议您喂母乳喂到宝宝几个月大？At what age of the child were you advised to continue breastfeeding to?（放到二维表里）	_____月month<br>888=没有建议喂到几个月did not specify month
9. 有人建议过您宝宝出生后应该立即喂母乳吗？Did you get advice on immediate breastfeeding after your child was born?	1=有yes<br>2=没有no
10. 有人建议过您喂配方奶吗？Were you advised to introduce formula?	1=有yes<br>2=没有no → 结束本单元 end of section
11. 谁向您建议过喂配方奶？【多选】Who advised you to introduce formula? [multiple choice]	1=村医 Village doctor<br>2=医院的医务人员Medical professional<br>3=宝宝的外婆Maternal grandmother<br>4=宝宝的奶奶Paternal grandmother<br>5=丈夫Husband<br>6=其他家庭成员，请注明：Other family member, specify: __________<br>7=朋友Friend<br>8=销售人员Salesperson<br>9=其他人，请注明Other, specify:____________
12. 你会听他们的嘛？（依据11生成二维表）<br>Would you listen to them?	1=会 Yes<br>2=不会 No
13. 这个人建议你喂配方奶喂到宝宝几个月大？At what age of the child were you advised to introduce formula?（放到二维表里）	___ 月months<br>888=没有建议喂到几个月did not specify month
14. 别人建议您喂配方奶的主要原因是什么？【不要读选项】What was the primary reason you were advised to introduce formula?	1=剖腹产C-section<br>2=肝炎hepatitis<br>3=阴道侧切术episiotomy<br>4=避免黄疸to avoid jaundice<br>5=宝宝不会饿到、哭得少Baby will not be hungry and will cry less<br>6=妈妈奶水不足I did not have enough breastmilk<br>7=配方奶和母乳一样好、甚至比母乳更好Formula is just as good or better than breastmilk<br>8=喂母乳不方便It is inconvenient<br>9=宝宝体重增加不足Baby will not gain enough weight<br>10=宝宝体重增加太多Baby will gain too much weight<br>11=其它，请注明Other, specify_____
15. 您曾经在哪里收到过配方奶粉的试用装？（可多选；一一读选项） From where have you received infant formula samples? [multiple choice; read choice one by one]	1=医院hospital/clinic<br>2=母婴店 baby store<br>3=超市 supermarket<br>4=朋友/邻居/亲戚赠送 gift from friends/neighbors/family<br>5=其他，请说明 other, specify_____<br>6=没有收到过 have never received any infant formula sample

D6. *喂养知识Knowledge

1.	出生后的第一口母乳对宝宝有害，应丢弃。你觉得正确吗？The first milk after birth is bad for your baby and should be discarded. Do you think it's right？	1=正确<br>0=不正确<br>999=不知道
2.	乳房小的妇女不能产足够的奶来喂宝宝。你觉得正确吗？Women with small breasts cannot produce enough milk to feed a baby. Do you think it's right？	1=正确<br>0=不正确<br>999=不知道
3.	除了母乳外，给六个月内的宝宝喂水也很重要。你觉得正确吗？It is important to feed your baby water in addition to breastmilk. Do you think it's right？	1=正确<br>0=不正确<br>999=不知道
4.	**母乳喂养的妈妈应比平时多喝水，以确保奶水充足。**你觉得正确吗？**Breastfeeding mothers should drink more than usual in order to ensure a good milk supply**	1=正确<br>0=不正确<br>999=不知道
5.	应该什么时候喂母乳？When should you breastfeed your child?	1=您吃饭前Right before you have a meal<br>2=您吃饭后Right after you finish a meal<br>**3=宝宝饿的时候Whenever he/she is hungry**<br>4=不论如何，每三个小时喂一次Every three hours, no matter what<br>999=不知道
6.	应该什么时候给宝宝开始喂水When should you start to introduce water to your child?	1=立即Immediately<br>2=出生一个月内Within the first month<br>**3=当宝宝开始吃固体食物的时候When he starts to eat solid foods**<br>4=当宝宝开始爬的时候When he starts to crawl<br>999=不知道
7.	应该什么时候给宝宝添加配方奶？When should you start to introduce formula to your child?	1=当宝宝3个月左右When he is around 3 months old<br>2=当宝宝6个月左右When he is around 6 months old<br>3=当宝宝1周岁左右When he is around 1 year old<br>**4=大部分宝宝不需要喝奶粉Most babies don't need to ever drink formula**<br>999=不知道
8.	应该什么时候给宝宝添加软的或者半固体的食物？When should you start to introduce soft or semi-solid foods to your child?	1=立即Right away<br>**2=当宝宝6个月的时候When he is around 6 months old**<br>3=当宝宝一周岁的时候When he is around 1 year old<br>4=当宝宝18 个月的时候When he is around 18 months old<br>999=不知道
9.	以下哪种食物是铁的最佳来源？Which of the following foods is the best source of iron?	**1=猪肉Pork**<br>2=蛋Egg<br>3=粥Porridge

	4=红薯Sweet potato<br>999=不知道
10. 了解宝宝是否贫血最好的方式是什么？What is the best way to know if your child has anemia?	1=宝宝看起来比同龄的孩子瘦小He looks smaller or skinnier than other babies his age<br>2=宝宝头发的颜色比较浅His hair looks lighter in color<br>**3=医生验血Blood test from doctor**<br>4=宝宝呼吸困难He has trouble breathing<br>999=不知道
11. 如果您感到悲伤或者负担过重，您认为以下哪一项是应对的好方法？If you are feeling sad or overwhelmed, which of the following is a good strategy for coping? （可多选）	1=自己解决，每个人都有烦心事Keep it to yourself, everyone has problems<br>**2=向朋友和家人寻求帮助Ask for help from friends and family**<br>3=多吃点Eat more<br>4=花更多时间在微信朋友圈上Spend more time on WeChat Moments<br>999=不知道
12. 防止宝宝感冒最佳的方式是什么？What is the best way to prevent your baby from getting a cold? （可多选）	**1=勤洗手Wash hands regularly**<br>2=尽量避免外出Limit trips outside of the house<br>3=用配方奶代替母乳Feed him formula instead of breastmilk<br>4=一直给宝宝戴着帽子Always have him wear a hat<br>999=不知道

D7. *喂养信息来源Feeding Information Sources

Reference:

- Alive & Thrive baseline survey: https://dataverse.harvard.edu/file.xhtml?persistentId=doi:10.7910/DVN/AORZAU/1RONMW&version=1.0

Related to feeding your child, where or to whom would you go to if you need advice with any of the following issues? Please choose top 2 sources and rank them based on how much you trust them. 以下是与宝宝喂养有关的问题，当您遇到以下问题需要寻求建议时，您会向谁寻求帮助？请选择您**最信任的人**或者信息来源。		
	Source 来源	Code
1. Problems you might encounter with feeding your child in the first week after the child was born宝宝出生后第一周喂养中可能遇到的问题		1=村医 village doc<br>2=医院人员 hospital staff<br>3=宝宝的妈妈child's mother<br>4=宝宝的爸爸child's dad<br>5=宝宝的奶奶child's paternal grandmother<br>6=宝宝的外婆child's maternal grandmother<br>7=其他家人other family members

2. Problems related to breastfeeding your child when the child is around 3 or 4 months of age宝宝3~4个月左右母乳喂养可能遇到的问题		8=邻居/朋友 neighbors/friends<br>9=育婴店店员 child supply store staff<br>10=上网查资料 online browsing<br>11=电视/广播 TV/radio<br>12=图书/报纸 book/news paper<br>13=微信群wechat group<br>14=无none<br>15=其他，请说明 other(specify)_____
3. How to introduce new soft, semi-solid or solid foods如何给宝宝添加新的食物		
4. how to choose infant formula怎么挑选配方奶粉		
5. Feeding when the child is sick宝宝生病时的喂养问题		
6. If the child has a poor appetite (doesn’t eat well for a few days)如果宝宝胃口不好（几天不好好吃东西）		
7. Do you have a smart phone? 您有智能手机吗？	1=yes是<br>2=no否→ skip to next section跳到下一部分	
8. 您的手机上有没有安装育儿软件？（可观察手机）	1=yes是<br>2=no否→ skip to Q10	
9. 您多久会看一次这些育儿软件？	1=从来不看<br>2=基本不看<br>3=有时候看<br>4=经常看<br>5=每天都看	
10. 您是否有关于养育宝宝的交流群？（微信或QQ）Does your village have a WeChat group for advice on raising your child? (WeChat or QQ)	1=有yes<br>2=没有no	

E. 疾病与医疗卫生服务Disease & Health Services

E1. 婴幼儿健康服务Newborn and Child Health Services

核实是否是目标样本：**该样本是0-6月龄宝宝吗？**<br>ELIGIBILITY CHECK: Is this a baby 0-6 months?<br>是Yes<br>否no → 跳过本单元skip this section	
1. 宝宝出生体重Child's weight at birth	克grams
2. 宝宝出生身长Child's birth length	厘米cm
3. 你在哪里生的宝宝？Where did you give birth?	1=村诊所 village clinic<br>2=私人诊所 Private clinic<br>3=乡镇卫生院 Township Health Center<br>4=县妇幼保健院 County Maternal and Child Health Hospital<br>5=县医院 County Hospital<br>6=市妇幼保健院 City Maternal and Child Health Hospital<br>7=市医院 City Hospital<br>8=职工医院 staff hospital<br>9=其他，请注明Other, please specify ______
4. 宝宝是第多少孕周出生的？At what gestational age was the baby born?	____周week
5. 宝宝生产方式？How was the child delivered?	1=自然分娩 natural vaginal Birth<br>2=辅助分娩（使用助产钳或吸引器）assisted vaginal birth (used forceps and/or suction)<br>3=剖宫（腹）产C-Section<br>9=不知道Don't know
6. 宝宝出院后接受过多少次体检（包括在家或者医院）？How many check-ups did your child have in total after being discharged from hospital (at home or clinics)?	___次 times
7. 宝宝出院后的头两个月接受过多少次体检（包括在家或者医院）？How many check-ups did your child have in the first 2 months after being discharged from hospital (at home or clinics)? （宝宝未满2月龄，就截止目前为止If younger than 2 months, ask "up till now"）	___次 times
8. 宝宝出院后10天内接受过多少次体检（包括在家或者医院）？How many check-ups did your child have in the first 10 days after being discharged from hospital (at home or clinics)? （宝宝未满10天，就截止目前为止If younger than 10 days, ask "up till now"）	___次 times

E2. 疾病与医疗Illness and Medical Care

- Reference: China Economic, population, nutrition, and health survey – 2011 Child Questionnaire

http://www.cpc.unc.edu/projects/china/data/questionnaires/C11child_Eng.pdf

- Alive & Thrive baseline survey: https://dataverse.harvard.edu/file.xhtml?persistentId=doi:10.7910/DVN/AORZAU/1RONMW&version=1.0
- Added a few questions based on Ann’s study in Madagascar

核实是否是目标样本：**该样本是0-6月龄宝宝吗？**<br>ELIGIBILITY CHECK: Is this a baby 0-6 months?<br>1=是yes<br>2=否no → 不做本单元skip this section		
In the past 2 weeks (including today), did your child have any of these symptoms?<br>在过去的两周内（包括今天），您的宝宝是否有下列症状？		
1. Fever 发烧	0=没有no → 下一症状next symptom<br><br>1=有yes<br><br>999=不知道unknown→ 下一症状skip to next symptom	您是否因为宝宝出现了这个症状，带宝宝去看了医生？Did this symptom prompt you to bring your child to a doctor/health professional?<br><br>0=没有no<br>1=有yes
2. cough咳嗽		
3. runny nose 流鼻涕		
4. Blood in the stool大便带血		
5. Diarrhea拉肚子		
6. Vomiting 呕吐		
7. Difficulty breathing 呼吸困难		
8. Skin rash, dermatitis 皮疹、皮炎		
9. Scrapes or cuts擦伤、割伤		
10. Bruising 淤伤		
11. Burns 烧伤		
12. Lethargy 没有精神		
13. Ear irritation耳部不适		
14. Eye irritation/redness 眼睛不适/发红		
15. Persistent crying 久哭不止		
如孩子过去两周无上述情况，跳至第21题<br>if the child did not have any symptom listed above, skip to Q21.		
16. How many times was your child seen by a doctor/health professional within the past two weeks? 你的宝宝在过去两周内看过几次医生？	____次times<br>If 0 → 第19题 Skip to Q19	
17. 你的宝宝上次是在哪里看医生的? Where did your child see a doctor last time?	1=村诊所Village clinic<br>2=私人诊所private clinic<br>3=乡镇卫生院Township hospital<br>4=县妇幼保健院 County Maternal and Child Health Hospital<br>5=县医院 County hospital<br>6=市妇幼保健院City Maternal and Child Health Hospital<br>7=市医院city hospital<br>8=职工医院Staff hospital<br>9=其他（请注明）other, specify<br>999=不知道 don’t know	
18. 医生在过去两周内给宝宝开药了吗Did any doctor/medical professional prescribe any medication to your child in the past 2 weeks?	1=有yes<br>2=没有no	
19. 除了医生在过去两周开的药之外，宝宝吃了其它药吗？Did your child take any medication other than those prescribed by a doctor/medical professional in the past 2 weeks?	1=吃了yes<br>2=没有吃no → skip to Q21	
20. 宝宝吃的其它药从哪里来的? Where did you get the medication?	1=从药店买的bought from pharmacies<br>2=之前看医生剩余的药leftover from previous doctor’s visit	

	3=其他 (请注明: _______)other (specify: _______)
21. 您是否曾通过网上平台向医生咨询有关宝宝身体健康的问题？Have you consulted medical doctors through online platforms about your child's health?	1=是yes<br>2=否no
22. 您是否曾通过网上平台向医生咨询有关你自己身体健康的问题？Have you consulted medical doctors through online platforms about your own health?	1=是yes<br>2=否no

E3. 影响婴幼儿健康的行为Behaviors Influencing Child Health

核实是否是目标样本：**该样本是0-6月龄宝宝吗？**<br>ELIGIBILITY CHECK: Is this a baby 0-6 months?<br>1=是yes → 回答全部 all questions<br>2=否no → 只**回答第1题和第2题 Q1-Q2 only**	
**1.** 回忆一下，您昨天什么时候洗手了？【不要读选项，可回答多项】Think about how you spent your day yesterday. When did you wash your hands? (do not read the options; choose all that apply)	1=早上第一件事就是洗手First thing in the morning<br>2=睡觉前Right before going to sleep<br>3=做饭前Before cooking<br>4=做饭后After cooking<br>5=吃饭前Before eating<br>6=吃饭后After eating<br>7=小便后After peeing<br>8=大便后After pooing<br>9=手看上去脏的时候When hands have visible dirt on them<br>10=照顾宝宝前before handling the baby<br>11=照顾宝宝后after handling the baby<br>12=给宝宝换尿布后after changing the baby's diapers<br>13=给宝宝擦屁股之后<br>14=其它，请注明Other, specify:_____
**2.** 通常情况下，您家怎么洗碗？In general, how does your family clean their dishes?	1=用水冲洗Rinse with water<br>2=用洗洁精清洗Wash with dish soap<br>3=紫外线消毒UV sanitization<br>4=其它，请注明Other, specify:______
3. 宝宝穿过尿布或尿不湿吗？Does your child ever wear diapers?	1=是Yes<br>2=否No (跳至第5题 skip to 5)
**4.** 宝宝一般什么时候穿尿布或尿不湿【多选】When does your child wear diapers? (multiple response question)	1=夜晚At night<br>2=出门When we leave the house<br>3=一直穿Always<br>4=其它，请注明Other, specify:______
5. 宝宝在不穿尿布或尿不湿的情况下，通常在哪里大便？Where does the baby usually deficate without a diaper?	0=从没发生过，一直穿尿布→ 结束本单元 This has never happened; always wear a diaper → end this section<br>1=在房间里，有专门的尿盆in a special basin for baby's pee/poo inside the house<br>2=在房间里，先拉在地板或地面上on the floor inside the house<br>3=房间内的厕所 in the toilet inside the house<br>4=房间外的厕所 in the toilet outside the house

	5=其他，请说明 other, specify________
6. 宝宝在不穿尿布或尿不湿的情况下，大便的频率是？How often does your child defecate outside his/her diaper, e.g., on the floor?	1=几乎每天Nearly every day<br>2=每周一次Once a week<br>3=每月一次Once a month<br>4=少于每月一次Less than once a month<br>5=从没发生过This has never happened (→结束本单元finished this section)
7. 宝宝在不穿尿布或尿不湿的情况下，您怎么清理宝宝的大便？【可多选】How do you clean after your child defecates outside his/her diaper (Multiple response questions)?	1=用干抹布、干拖把或干纸巾擦掉Wipe it with a dry cloth, mop, or paper<br>2=用湿抹布、湿拖把或湿纸巾擦掉Wipe it with a wet cloth, mop, or paper<br>3=用沙子/土等盖住然后扫进垃圾桶Sprinkle sand on it and sweep it into a bin<br>4=其它，请注明Other, specify:____
8. 之后，您怎么清理被大便弄脏的地面或桌面等地方？Do you sanitize the surface in any way?	1=不清洁No<br>2=会，只用水 yes, only water<br>3=会，用肥皂Yes, with soap<br>4=会，用酒精或含漂白成分的清洗剂Yes, with an alcohol or bleach-based cleaner<br>5=其他，请注明other, specify______
9. 之后，您会洗手吗？Do you wash your hands afterwards?	1=会yes<br>2=不会no
10. 给宝宝准备或喂食物之前您洗手的频率？How frequent do you wash your hand when you feed your baby?	1=从不Never<br>2=很少Rarely<br>3=经常Often<br>4=总是Always
11. 给宝宝擦了屁股之后您洗手的频率？How often do you wash your hands after cleaning your child's bottom?	1=从不Never<br>2=很少Rarely<br>3=经常Often<br>4=总是Always

E4. 妈妈/孕妇保健Perinatal Care

核实是否为目标样本：目标样本是孕妇或者0-6个月龄宝宝的妈妈吗？<br>ELIGIBILITY CHECK: Is the respondent a pregnant woman or mother of children 0 – 6 months of age?<br><br>1=孕妇pregnant woman → 回答1-10题 answer question 1 – 10<br>2=0-6月龄宝宝的妈妈 mother of baby 0-6 months → 回答所有题answer all questions<br>3=否 → 不做本单元skip this section	
请就您本次怀孕（对于孕妇）或者最近一次怀孕（对于妈妈）的经历回答以下问题。Answer the following questions based on your current pregnancy (for pregnant woman) or most recent pregnancy (for mother of child).	
1. 在怀孕期间，你服用过叶酸吗？ During your pregnancy, did you take any folic acid?	1=是 Yes<br>2=否 No --> skip to Q3<br>999=不知道 Don't know
2. 怀孕期间你吃了多少天叶酸？how many days did you take folic acid during pregnancy	____ 天days
3. 您领取过免费的叶酸吗？Have you received any free folic acid?	1=是 Yes<br>2=否 No
4. 在怀孕期间，您服用过铁补充剂吗？During your pregnancy, did you take any iron supplements?	1=是 Yes<br>2=否 No

	999=不知道 Don’t know
5. 怀孕期间你吃了多少天铁补充剂？how many days did you take iron supplements during pregnancy?	____ 天days
6. 在怀孕期间，您服用过钙补充剂吗？During your pregnancy, did you take any calcium supplements?	1=是 Yes<br>2=否 No<br>999=不知道 Don’t know
7. 在怀孕期间，您服用过其他微量元素补充剂吗？During your pregnancy, did you take any other micro-nutrient supplements?	1=是 Yes，请说明______<br>2=否 No<br>999=不知道 Don’t know
8. 您在怀孕期间是否有以下妊娠并发症【逐一问除了“没有”之外的选项，可多选】？ Did you have any of the following pregnancy complications during your pregnancy?[ask about each option one by one, except for “none”]	1=孕期高血压gestational high blood pressure<br>2=孕期糖尿病gestational diabetes<br>3=贫血anemia<br>4=其它, 注明other, specify____<br>0=没有none
9. 您被诊断出患有肝炎吗？Have you ever been diagnosed with hepatitis?	1=有 yes<br>2=没有 no
10. 在您怀孕期间，医务人员是否到过您家家访？During your pregnancy, did any medical provider visit you at home?	1=是Yes<br>2=否No
11. 怀着这个宝宝的时候（妈妈）是否进行过产前检查？When you were pregnant, have you had any prenatal visit at the hospital/clinic?	1=yes是<br>2=no 否→ skip to Q14跳至14题
12. 第一次产检时，您怀孕几个月How many months pregnant were you the first time you went for your prenatal visit?	_______月 Number of month
13. 您总共进行了几次产前检查？How many prenatal visits did you have altogether?	____次times
14. 在您生完孩子后，医院里的医务人员是否给您做过体检？Did any medical staff give you a medical examination in the hospital after you gave birth?	1=是Yes<br>2=否No
15. 在您生完孩子出院后，医务人员是否给您做过体检？Did any medical staff give you a medical examination after you gave birth and left the hospital?	1=是Yes<br>2=否No → 结束本单元 end of section
16. 您在哪些地点进行过体检？【多选】where did the exam take place? [multiple choice]	1=在家at home<br>2=县级医院及县级以上医院county-level houspital or above<br>3=镇级医院township-level hospital<br>4=村诊所village clinic<br>5=其它，注明other, specify_____

E5. 妈妈/孕妇营养Perinatal Nutrition

核实是否为目标样本：该样本是孕妇或正在哺乳的妈妈吗？Is the respondent pregnant now or breastfeeding?

1=孕妇pregnant woman → 回答1-17题 answer question 1 - 17

2=正在哺乳的妈妈 Breastfeeding mom → 回答所有题answer all questions

3=否 No → 不做本单元skip this section

请根据您昨天（包括白天和晚上）的饮食情况回答1-17问题。Please answer the questions 1-17 based on the foods you ate yesterday, during the day and night.

1. Did you eat any staple food yesterday, such as rice porridge, flour porridge, steamed bun or rice您昨天是否吃了米汤、粥、面汤、馒头或米饭等主食类食物？	1=是yes<br>2=否no<br>999=不知道don't know
2. Did you eat any yellow or orange food yesterday, such as pumpkin, carrot or red sweet potato您昨天是否吃了南瓜、胡萝卜、红心红薯等里面是黄色或橙色的食物	
3. Did you eat any root and stem vegetables yesterday, such as potato, yam, radish, white sweet potato您昨天是否吃了土豆、山药、白萝 卜、白心红薯等根茎类食物？	
4. Did you eat any leafy dark green vegetables yesterday? 您昨天是否吃了深绿色且有叶子的蔬菜？	
5. Did you eat any red or yellow fruits such as persimmon, apricot, watermelon, cantaloupe or tomato yesterday? 您昨天是否吃了红或黄色的水果，如柿子、杏、西瓜、哈密瓜或番茄?	
6. Did you eat any other fruits or vegetables yesterday? 您昨天是否吃了其他水果或蔬菜?	
7. Did you eat any organ meats such as animal liver, kidney or heart yesterday? 您昨天是否吃了内脏类食物，如动物肝、肾或心脏	
8. Did you eat any meat or meat products (e.g., chicken, duck, pork, beef, lamb, etc.) yesterday? 您昨天是否吃了肉类或肉类产品（如鸡肉、鸭肉、猪肉、牛肉、羊肉等）	
9. Did you eat any eggs yesterday? 您昨天是否吃了鸡蛋	
10. Did you eat any fresh or dried fish, shellfish or seafood yesterday? 您昨天是否吃了鱼类、贝类或海鲜类食物?	
11. Did you eat any beans, peas, lentils, nuts or seeds yesterday? Please note: Drinking soy milk counts. 您昨天是否吃了扁豆等豆类、豆制品或坚果、种子? 注意：喝过豆浆算吃过豆制品	
12. 您昨天有没有喝动物奶（比如牛奶、羊奶）或动物奶粉？Did you have any milk (such as cow or sheep milk) or powdered milk?	
13. Did you eat any dairy products such as cheese and yoghurt yesterday?您昨天是否吃了乳制品，如奶酪和酸奶	
14. Did you eat any oil (including cooking oil) and meat fat yesterday?您昨天是否吃了油（包括炒菜用的油）、肥肉（含肉汤）等脂肪类的食物	
15. Did you eat any snacks such as biscuit, dessert, candy, chocolate and cake yesterday? 您昨天是否吃了零食，如饼干，甜点，糖果，巧克力和蛋糕	
16. 您昨天是否服用了含铁的微量元素补充剂？Did you take any micronutrient supplement with iron?	
17. 您昨天是否喝了酒？Did you drink any alcohol yesterday?	
18. 【使用道具】我想问一下您在喂母乳期间的饮水量。如果用这个杯子来装，您每天大概喝几杯水（包括茶）？[using prop] I would like to ask about your water intake during breastfeeding. Using this cup as a measure, how many cups of water do you drink in a day on average (including tea)?	___杯cups
19. 【使用道具】如果用这个杯子来装，您每天大概喝几杯汤？[using prop] Using this cup as a measure, how many cups of soup do you drink in a day on average?	___杯cups

F. 怀孕史Pregnancy History

检查是否为目标样本：该目标样本是孕妇或0-6月龄宝宝的母亲？

Check target sample: The target sample is a pregnant woman or 0-6 months child's mother.

1=是yes 2=否no → Skip to the next section

问题 Questions	答案 Anwers
1. 你在这次怀孕（孕妇）/生这个宝宝<u>之前</u>怀孕过几次？<br>How many times have you been pregnant, **<u>excluding</u>** the current one for pregnant women and the most recent one for mothers?	______次<br><br>如无，跳到下一部分。<br>If 0, → skip this section.
2. 这其中一共有几次活产婴儿？<br>Among them, how many of them are live-birth?	______次
3. 在活产婴儿中，有几个低体重出生的宝宝（低于2500克/5斤）？<br>How many of live-births have low birth weight (lower than 2500g)?	______个

G. 心理健康Psychological Well-Being

G1. *抑郁、焦虑与压力 Depression, Anxiety, and Stress Scales (DASS-21)

根本不符合	有时候符合	基本符合	非常符合
**0**	**1**	**2**	**3**

请根据过去<u>一周之内</u>符合您的实际情况，选择相应的数字（0，1，2 或3）。您的回答没有对错之分，所以请不要在以下任何一项上花太多时间。Please select a number 0, 1, 2 or 3 which indicates how much the statement applied to you over the past week. There are no right or wrong answers. Do not spend too much time on any one statement.

在过去七天 in the past 7 days	
1. 我发现很难让自己安静下来休息I found it hard to wind down?	0=根本不符合did not apply to me at all<br>1=有时候符合applied to me to some degree or for some of the time<br>2=基本符合applied to me to a considerable degree or for a good part of time<br>3=非常符合applied to me very much or most of the time
2. 我感到嘴巴很干I was aware of dryness of my mouth	
3. 我似乎完全不能积极乐观起来I couldn't seem to experience any positive feeling at all	
4. 我感到过呼吸困难（例如： 在没有体力透支的情况下而感到呼吸急促，喘不过气来）I experienced breathing difficulty (e.g. excessively rapid breathing, breathlessness in the absence of physical exertion)	
5. 我似乎没法提起劲来做事情I found it difficult to work up the initiative to do things	
6. 在面对一些情况时我容易反应过度I tended to over-react to situation	
7. 我曾发抖（例如：手打哆嗦）I experienced trembling (e.g. in the hands)	
8. 我感到时常神经紧张I felt that I was using a lot of nervous energy	
9. 我担心自己可能因为惊慌而干蠢事出洋相I was worried about situations in which I might panic and make a fool of myself	
10. 我感到我没什么可期待的I felt that I had nothing to look forward to	

11. 我发现自己变得心焦 I found myself getting agitated	
12. 我发现很难放松下来 I found it difficult to relax	
13. 我感到消沉和沮丧 I felt down-hearted and blue	
14. 我无法容忍我正在做的事情被打断I was intolerant of anything that kept me from getting on with what I was doing	
15. 我感到我我快要恐慌I felt I was close to panic	
16. 我对任何事情都没法充满热情 I was unable to become enthusiastic about anything	
17. 我曾感到自己没有存在的价值I felt I wasn't worth much as a person	
18. 我感到我曾极容易因为小事而生气 I felt that I was rather touchy	
19. 在没有体力透支的情况下我也能感觉到自己的心跳或心律不正常（例如：感到心跳过快或心律不齐）I was aware of the action of my heart in the absence of physical exertion (e.g. sense of heart rate increase, heart missing a beat)	
20. 没有什么特殊原因的情况下，我感到害怕 I felt scared without any good reason	
21. 我感到生命没有价值 I felt that life was meaningless	

G2. 孕产妇抑郁 perinatal depression

Einburgh Postpartun Depression Scale

核实是否为目标样本：目标样本是孕妇或者0-6月龄宝宝的妈妈吗？<br>ELIGIBILITY CHECK: Is the respondent a pregnant woman or mother of children 0 – 6 months of age?<br>1. Yes是<br>2. No ➔ skip to next section否➔ 跳到下一部分	
你正怀着宝宝或者最近生了宝宝，我们想了解一下您的感受，请根据您过去七天的感受选择答案，而不仅仅是今天。As you are pregnant or have recently had a child, we would like to know how you are feeling. Please choose the answer that comes closest to how you have felt IN THE PAST 7 DAYS, not just how you feel today.	
在过去七天 In the past 7 days … 【读选项 Read the following】	选项 Options
1. 我能看到事情有趣的一面，并笑得开心 I have been able to laugh and see the funny side of things as much as I always could.	0=和以前一样As much as I always could<br>1=比以前少一些Not quite so much now.<br>2=肯定比以前少 Definitely not so much now<br>3=完全做不到 Not at all
2. 我对未来有所期待。 I have looked forward with enjoyment to things.	0=和以前一样 As much as I ever did<br>1=比以前少一些Rather less than I used to<br>2=肯定比以前少Definitely less than I used to<br>3=完全做不到Hardly at all
3. 当事情出错时，我会过分得责备自己 I have blamed myself unnecessarily when things went wrong.	3=大部分时候这样Yes, most of the time.<br>2=有時候这样Yes, some of the time<br>1=不经常这样Not very often<br>0=沒有这样No, never
4. 我会无缘无故感到焦虑和担心 I have been anxious or worried for no good reasons.	0=一点也沒有No, not at all.<br>1=极少有Hardly, ever<br>2=有时候这样 Yes, sometimes

	3=经常这样Yes, very often
5. 我无缘无故感到害怕和惊慌 I have felt scared or panicky for no very good reason.	3=大部分时候这样样Yes, quite a lot<br>2=有时候这样Yes, sometimes<br>1=不经常这样No, not much<br>0=沒有这样No, not at all
6. 当很多事情冲着我来，使我透不过气Things have been getting on top of me.	3=大多数时候不能应付 Yes, most of the time I haven't been able to cope at all<br>2=有时不能应付Yes, sometimes I haven't been coping as well as usual<br>1=基本可以应付No, most of the time I have coped quite will<br>0=一直可以应付No, I have been coping as well as ever
7. 我很不开心，以致失眠 I have been so unhappy that I have had difficulty sleeping	3=经常这样Yes, most of the time<br>2=有时这样Yes, sometimes<br>1=偶尔这样Not very often<br>0=沒有这样No, not at all
8. 我感到难过或悲伤 I have felt sad or miserable.	3=经常这样Yes, most of the time<br>2=有时这样Yes, quite often<br>1=偶尔这样Not very often<br>0=沒有这样No, not at all
9. 我不开心到哭 I have been so unhappy that I have been crying.	3=经常这样Yes, most of the time<br>2=有时这样Yes, quite often<br>1=偶尔这样Not very often<br>0=沒有这样No, not at all
10. 我想过要伤害自己 The thought of harming myself has occurred to me.	3=经常这样 Yes, quite often<br>2=有时候这样Sometimes<br>1=很少这样 Hardly ever<br>0=从来沒有Never

H. *决策力Decision-Making

- Shroff MR, Griffiths PL, Suchindran C, Nagalla B, Vazir S, Bentley ME. Does maternal autonomy influence feeding practices and infant growth in rural India? *Social Science & Medicine.* 2011;73(3):447-455.
    - Adapted questions from 2 sections: household decision-making & childcare decision-making autonomy
- Peterman, Amber and Schwab, Benjamin and Roy, Shalini and Hidrobo, Melissa and Gilligan, Daniel O., Measuring Women's Decisionmaking: Indicator Choice and Survey Design Experiments from Cash and Food Transfer Evaluations in Ecuador, Uganda, and Yemen. IFPRI Discussion Paper 1453.
    - Added the question on disagreement and a few other decisions

	a. 在您家里，通常是由谁来决定……？ Who in your family usually has the final say on the decision?<br><br>1=受访者本人Respondent<br>2=与家庭中的其他人共同决	b. 在过去一个月里，您和家人在这个问题上有过不同意见吗？In the last month, has there been a disagreement about this type of decisions?

	定Jointly with others in household<br>3=家庭中的其他人Others in the household	1=有 yes<br>2=没有 no<br>3= 过去一个月里这个问题没有出现过this decision has not come up in the last month
1. 家里吃饭要买什么食物 What food to buy for family meals?		
2. 宝宝妈妈是否应该工作赚钱 Whether or not the child's mother should work to earn money?		
3. 是否购买大件商品，如车子 Whether to purchase major goods for the household such as a TV?		
4. 家庭的收入怎么花How the child's father's earnings are spent?		
5. 是否在宝宝满6个月前进行纯母乳喂养？Exclusively breastfeed newborn for 6 months?		
6. 给宝宝吃什么食物What foods to feed the child?		
7. 宝宝生病了怎么办What to do if the child falls sick?		
8. 在宝宝医疗上花多少钱？How much to spent on health care for the child?		
9. 【观察题】在问这些问题的过程中，被访者身边有其他家庭成员吗Were there other household members aside from the respondent present during these questions?	1=只有被访者一人Respondent was alone<br>2=有其他家庭成员在场Other household members were present	

I. *社会支持Perceived Social Support

社会支持Multidimensional Scale of Perceived Social Support

- Original: Zimet GD, Dahlem NW, Zimet SG, Farley GK. The Multidimensional Scale of Perceived Social Support. Journal of Personality Assessment 1988;52:30-41.
- Validation/use in China:
    - Wang J, Miller JK, Zhao X. Family Functioning and Social Support in Men and Women Diagnosed with Depression in China. Contemp Fam Ther. 2014;36(2):232-241. doi:10.1007/s10591-013-9294-y
    - Wang Y, Wan Q, Huang Z, Huang L, Kong F. Psychometric properties of multi-dimensional scale of perceived social support in Chinese parents of children with cerebral palsy. Front Psychol. 2017;8(NOV):2020. doi:10.3389/fpsyg.2017.02020

非常不符合	不符合	稍不符合	中立	稍符合	符合	非常符合
1	2	3	4	5	6	7

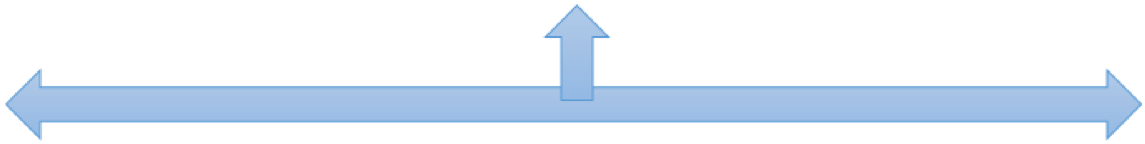

<table>
<tr><td colspan="2">填答说明：以下12个句子，每一个句子后面各有7个答案，测试您的社会支持程度。请您根据自己的实际情况在每句后面选择一个答案。例如，选择1表示该描述与您的实际情况非常不符合，选择7表示该描述与您的实际情况非常符合。
Instructions: We are interested in how you feel about the following statements. Listen to each statement carefully. Indicate how you feel about each statement.</td></tr>
<tr><td>1. 在我遇到问题时，我生活中有个特殊的人(比如家人、朋友或同学)会出现在我的身旁。There is a special person who is around when I am in need</td><td rowspan="12">1=非常不符合very strongly disagree
2=不符合strongly disagree
3=稍不符合mildly disagree
4=中立neutral
5=稍符合mildly agree
6=符合strongly agree
7=非常符合very strongly agree</td></tr>
<tr><td>2. 我生活中有一个特殊的人（比如家人、朋友或同学），我可以与他/她共享快乐与忧伤。There is a special person with whom I can share joys and sorrows.</td></tr>
<tr><td>3. 我的家庭能够真正地给我帮助。My family really tries to help me.</td></tr>
<tr><td>4. 在需要时我能够从家庭获得感情上的帮助和支持。I get the emotional help & support I need from my family.</td></tr>
<tr><td>5. 我生活中有个特殊的人（比如家人、朋友或是同学）总能真正地安慰我 I have a special person who is a real source of comfort to me.</td></tr>
<tr><td>6. 我的朋友们会尽力帮助我。My friends really try to help me.</td></tr>
<tr><td>7. 在发生困难时我可以依靠我的朋友们。I can count on my friends when things go wrong.</td></tr>
<tr><td>8. 我能与自己的家庭谈论我的难题。I can talk about my problem with my family.</td></tr>
<tr><td>9. 我的朋友们能与我分享快乐与忧伤。I have friends with whom I can share my joys and sorrows</td></tr>
<tr><td>10. 在我的生活中有个特殊的人（比如家人、朋友或同学）关心着我的感受。There is a special person in my life who cares about my feelings.</td></tr>
<tr><td>11. 我的家人乐意协助我作出各种决定。My family is willing to help me make decisions.</td></tr>
<tr><td>12. 我能与朋友们讨论自己的难题。I can talk about my problems with my friends.</td></tr>
</table>

J. 育儿开销 Child Expenses

核实是否是目标样本：**该样本是0-6月龄宝宝吗？**

ELIGIBILITY CHECK: Is this a baby 0-6 months?

1=是 yes　　　　2=否 no → skip this section

1. How much did you and your family spend on snacks for your child in the past month? 您家最近一个月给宝宝购买零食大概花费了多少钱？	____ 元 RMB
2. How much did your family spend on food in the past month? 您家最近一个月买菜大概花费了多少钱？	
3. How much did you and your family spend on micronutrient supplements for your child in the past month? 您家最近一个月给宝宝购买微量元素补充剂大概花费了多少	
4. How much did you and your family spend on alcohol and cigrettes in the past month? 您家最近一个月买烟酒大概花了多少钱？	
5. How much did you and your family spend on gifts for friends and family in the past month? 您家最近一个月给亲戚朋友送礼大概花了多少钱？	
6. How many cans of formula did your child consume in the past month? 您孩子最近一个月喝了多少罐奶粉？	___ units罐<br>如果0罐奶粉if 0, 结束本单元<br>→ end of section
7. What was the price for the formula you bought last time? 您上次购买的奶粉每罐多少钱？	____ 元 RMB
8. How much did you spend on formula in the past month? 您上个月给孩子买奶粉一共花了多少钱？	____ 元 RMB

K.　养育人社会网络调查表 The social network of the caregivers

调查员：“请您回想一下，最近半年（2019年5月至今），您是否与人交流了有关养育孩子的信息？”（先列出所有人的名字，至多列出5个，再逐一访问）

Enumerator: “Over the past half year (from May 2019), have you ever communicated with someone about how to raise a child? ” (List all the names first up to 5, then interview one by one)

【填表说明】Instructions

（1）　角色: 1=村医，2=村妇女主任，3=村其他干部，4=同村村民，5=外村人，6=乡镇医生，7=其他请说明

Role: 1=village doctor, 2=village woman officer, 3=other officials from the village, 4=other villagers, 5=people outside of the village, 6=doctors from the township health center, 7=others, please specify

（2）　交流频次：1=少于每月交流一次， 2=每月交流一次以上，3=每周交流一次以上，4=每天交流一次以上；

Frequency of communication: 1=less than once every month; 2=more than once every month, 3=more than once every week, 4=more than once everyday

（3）　信任程度：1-5分；

Trust degree: 1-5

（4）　亲密程度：1-5分；

Intimacy：　1-5

交流人姓名 Who did you communicate (name)	角色 Role （1）	交流频次 Frequency （2）	信任程度 Trust degree（3）	亲密程度 Intimacy（4）	你以后还会与他/她交流养孩子的事情吗？ Will you communicate with him/her about how to raise a child?	当你心情不好的时候会不会向她倾诉？ Would you go to him/her when you feel sad?

【最后】如果以后每个月有健康管理员来你家里，教你如何养育孩子，你愿意吗？

If there is a community health worker coming to home once a month to distribute curriculums on how to raise babies, would you be interested?

1=愿意 Yes　2=不愿意 No

L. 体检结果 Physical Exam Results

L1. 孕妇/妈妈 Pregnant Woman/Mother of Infant

1. 受试者编码 Respondent ID	____
2. 受试者是孕妇还是妈妈？Is the respondent a pregnant woman or mother of infant?	1=孕妇pregnant woman<br>2=妈妈（不测血红蛋白）mother of infant (no hemoglobin test) → skip to Q7
3. 受试者完成了血红蛋白测定吗？Did the respondent complete the hemoglobin test?	1=是yes → skip to Q5<br>2=否no
4. 为什么受试者没有完成血红蛋白测定？What was the reason for not completing the hemoglobin test?	1=受试者认为测量不安全 respondent did not think it is safe to get tested<br>2=受试者拒绝或不愿意完成测量respondent refused or was not willing to complete the test<br>3=受试者尝试但未能完成测量 respondent tried but could not complete the test<br>4=受试者不明白测量方法 respondent did not understand the testing method<br>5=没有合适的空间进行测量 did not have the appropriate space to perform the test<br>6=设备问题 issues with testing machine<br>7=其它（请注明）____other (please specify)<br><br>→ 跳至skip to Q7
5. 血红蛋白值Hemoglobin	_____mg/L
6. 检测结果是第几次采血? Which blood collection did the hemoglobin result come from?	_____ 次times
7. 受试者完成了身高测定吗？ Did the respondent complete the height test?	1=是yes → skip to Q9<br>2=否no
8. 为什么受试者没有完成身高测定？What was the reason for not completing the height test?	1=受试者认为测量不安全 respondent did not think it is safe to get tested<br>2=受试者拒绝或不愿意完成测量respondent refused or was not willing to complete the test<br>3=受试者尝试但未能完成测量 respondent tried but could not complete the test<br>4=受试者不明白测量方法 respondent did not understand the testing method<br>5=没有合适的空间进行测量 did not have the appropriate space to perform the test<br>6=设备问题 issues with testing machine<br>7=其它（请注明）____other (please specify)<br><br>→ 跳至skip to Q10
9. 身高Height	_____厘米centimeter
10. 受试者完成了体重测定吗？ Did the respondent complete the weight test?	1=是yes → 跳至Q12 skip to Q12<br>2=否no
11. 为什么受试者没有完成体重测定？What was the reason for not completing the weight test?	1=受试者认为测量不安全 respondent did not think it is safe to get tested<br>2=受试者拒绝或不愿意完成测量respondent refused or was not willing to complete the test

	3=受试者尝试但未能完成测量 respondent tried but could not complete the test<br>4=受试者不明白测量方法 respondent did not understand the testing method<br>5=没有合适的空间进行测量 did not have the appropriate space to perform the test<br>6=设备问题 issues with testing machine<br>7=其它（请注明）____other (please specify)<br><br>→ 跳至skip to Q13
12. 体重Weight	_____公斤kilo
13. 体检护士姓名examiner name	_____
14. 体检日期、时间date & time	2019年year___月month___日day<br>___午 AM/PM___点hour

L2. 婴幼儿Infant

1. 受试者完成了体重测定吗？ Did the respondent complete the weight test?	1=是yes → skip to 3<br>2=否no
2. 为什么受试者没有完成体重测定？What was the reason for not completing the weight test?	1=家长认为测量不安全 caregiver did not think it is safe to get tested<br>2=家长拒绝或不愿意完成测量caregiver refused or was not willing to complete the test<br>3=受试者未能完成测量 respondent could not complete the test<br>4=家长不明白测量方法 caregiver did not understand the testing method<br>5=没有合适的空间进行测量 did not have the appropriate space to perform the test<br>6=设备问题 issues with testing machine<br>7=其它（请注明）____other (please specify)<br><br>→ 跳至Skip to Q4
3. 体重（净重）Weight (net)	_____公斤kilo
4. 受试者完成了身高测定吗？ Did the respondent complete the height test?	1=是yes → 跳至Q6 skip to Q6<br>2=否no
5. 为什么受试者没有完成身高测定？What was the reason for not completing the height test?	1=家长认为测量不安全 caregiver did not think it is safe to get tested<br>2=家长拒绝或不愿意完成测量caregiver refused or was not willing to complete the test<br>3=受试者未能完成测量 respondent could not complete the test<br>4=家长不明白测量方法 caregiver did not understand the testing method<br>5=没有合适的空间进行测量 did not have the appropriate space to perform the test<br>6=设备问题 issues with testing machine<br>7=其它（请注明）____other (please specify)<br><br>→ 跳至skip to Q7
6. 身高/长Height/length	_____厘米centimeter

7.	受试者完成了血红蛋白测定吗？Did the respondent complete the hemoglobin test?	1=是yes → 跳至skip to Q9<br>2=否no
8.	为什么受试者没有完成血红蛋白测定？What was the reason for not completing the hemoglobin test?	1=家长认为测量不安全 caregiver did not think it is safe to get tested<br>2=家长拒绝或不愿意完成测量caregiver refused or was not willing to complete the test<br>3=受试者未能完成测量 respondent could not complete the test<br>4=家长不明白测量方法 caregiver did not understand the testing method<br>5=没有合适的空间进行测量 did not have the appropriate space to perform the test<br>6=设备问题 issues with testing machine<br>7=其它（请注明）____other (please specify)<br>→ 跳至skip to Q11
9.	血红蛋白值Hemoglobin	_____mg/L
10.	检测结果是第几次采血? Which blood collection did the hemoglobin result come from?	第_____次time
11.	体检护士姓名examiner name	_____
12.	体检日期、时间date & time	2019年year___月month___日day<br>___午 AM/PM___点hour

设备编码及环境情况

1.	海拔 altitude	_____米（m）
2.	气温 temperature	_____℃
3.	气压 air pressure	_____帕（Pa）
4.	气压-海拔仪编号 air pressure-altitude machine No.	
5.	血红蛋白检测机器编号Hemoglobin machine No.	

FOLLOW-UP SURVEY

健康未来 - 新冠跟进调研问卷
Healthy Future COVID Follow-up Survey

目录 Table of Contents

目录 Table of Contents 62
开场白Enumerator self-introduction..64
表头Header 64
A. 基本信息 Basic Information 65
B. 父母外出情况 Parental Migration Status..65
C. 健康医疗服务 Health Care Seeking and Delivery Methods 66
C1. 婴幼儿健康服务 Newborn and Child Health Services.. 66
C2. 妈妈/孕妇保健 Perinatal Care 68

C3. 影响婴幼儿健康的行为 Behaviors Influencing Child Health 69
D. 喂养行为 Nutrition and Feeding Behavior...69
D1. 婴幼儿喂养习惯 Breastfeeding and IYCF Practice.. 69
F. 新冠肺炎影响 COVID-19 74
F1. 新冠肺炎风险感知 COVID-19 Risk Perception 74
F2. 新冠肺炎症状与感染COVID-19 Symptoms and infections 77
G. 母亲心理健康 Maternal Mental Health 78
H. 开销 Expenditures 80

开场白Enumerator self-introduction

您好！我是四川大学华西公共卫生学院在校学生，现在在做一个社会调研。几个月以前，我们来过您家，问了一些关于您（和您的宝宝）健康的问题，您还记得吧？今天我们想和您再做一下访谈，了解一下目前的情况，大概会占用您20-30分钟的时间。您所有的回答都会保密，您也可以随时决定结束访谈。感谢您的配合！

Hello! I am a student from Sichuan University school of public health, currently doing a social survey. A few months ago, we came to your home to ask a few questions about your (and your baby's) health – do you remember? Today, we would like to interview you again to learn about your current situation; it will take about 20-30 minutes of your time. All your answers will be anonymous, and you can decline the interview at any time. Thank you for your cooperation!

表头Header

【如果样本是婴幼儿且与母亲同住，第一养育人是婴幼儿的母亲】 If a sample baby lives with his/her mother, the mother is the primary caregiver by default.

【访谈员确认第一养育人是否发生变化。如果第一养育人发生变化，此次访谈对象为新的第一养育人。】 The enumerator should confirm if the primary caregiver of the baby has changed. If so, this survey is supposed to be delivered to the new primary caregiver.

第一养育人发生变化的情况包括但不限于 The conditions where the primary caregiver has changed include, but not limited to the following scenarios：

1） 基线时母亲与婴幼儿同住，现在不再与婴幼儿同住，新的第一养育人为宝宝的主要照顾人。If the mother lived with the baby at the baseline, but does not live with the baby now, the new primary caregiver is the person in the household primarily responsible for the baby's care.
2） 基线时母亲与婴幼儿不同住，现在与婴幼儿同住，新的第一养育人为宝宝的母亲 If the mother did not live with the baby at the baseline, but lives with the baby now, the primary caregiver is the baby's mother.
3） 其它第一养育人更换的情况，与各队队长确认。For other conditions, please confirm with your team supervisor.

【确认被访者目前是样本的孕妇或宝宝的主要照顾人。如果被访者不是孕妇或宝宝主要照顾人，向对方索要孕妇或宝宝主要照顾人的联系方式，或者约定时间通话，并结束电话】 Confirm respondent is pregnant woman or primary caregiver of a sample baby. If the respondent is not pregnant women or baby's primary caregiver, get contact information for the pregnant woman/primary caregiver or schedule a time to speak with that person, then end the call.

调查日期 Date of investigation
调查员编码 Investigator ID
调查员姓名 Name of investigator
县 County

镇 Township
村 Village
组/自然村 Group/Natural Village
家庭编码 Family code
第一养育人是否发生改变？Has the primary caregiver changed?<br>1.改变 Yes 2. 没有改变 No →下一部分 Next section<br><br>【备注：只要母亲与婴幼儿同住，第一养育人即为母亲】As long as the mother lives with the sample baby, the mother is the primary caregiver.
第一养育人的姓名 Primary Caregiver's name
第一养育人与宝宝的关系 Relationship between Primary Caregiver and Baby
户主姓名 Household Head Name

A. 基本信息 Basic Information

【逐一完成网上问卷的问题，同时使用链接记录答案】Record responses to each question one-by-one in Survey Solutions form.

**核实是否为目标样本：目标样本的生产日或预产期是在2019年11月12日以后吗？**<br>**ELIGIBILITY CHECK: Is the index a baby whose (expected) birth date was after November 12, 2019 during the baseline?**<br>1=是 Yes → 回答所有题answer all questions<br>2=否 No → 核实宝宝的生产日和性别并结束本单元 Check the birthdate and the baby's gender and go to next section	
1. 宝宝出生的年月日或预产期<br>(阳历；举例，2019年1月1日应写成20190101）？<br>The child's (expected) birth date (western calendar)?	____年 year<br>____月 month<br>____日day
2. 宝宝是否出生? Has the child been born yet?	1=是 Yes<br>2=否 No→ End of Section
3. 宝宝的性别? child's sex?	1=男 boy 2=女 girl

B. 父母外出情况 Parental Migration Status

**核实是否为目标样本：宝宝出生了吗？**<br>**ELIGIBILITY CHECK: Has the baby been born yet?**<br>1=是 Yes -> 回答所有问题 answer all questions<br>2=否 No -> 结束本单元 Skip the section	
1. 宝宝的妈妈现在是否和宝宝住在一起？Does the mother live with the child?	1=是Yes→Skip to Q3 跳至第3题<br>2=否No

2. 宝宝的妈妈在宝宝几个月大的时候离开? How old was the child when the mother left?	____月 months
3. 宝宝的爸爸现在是否和宝宝住在一起？Does the father live with the child?	1=是Yes→ End of the Section 结束本部分<br>2=否No
4. 宝宝的爸爸在宝宝几个月大的时候离开?How old was the child when the father left?	____月 months

C. 健康医疗服务 Health Care Seeking and Delivery Methods

C1. 婴幼儿健康服务 Newborn and Child Health Services

**核实是否是目标样本：该样本是在2019年11月12日以后出生的宝宝吗？**<br>**ELIGIBILITY CHECK: Is this a baby who was born after November 12, 2019?**<br>1 = 是 Yes → 回答所有问题 Answer questions from this section<br>2 = 2019年11月12日以前出生的宝宝 Born before November 12, 2019 → 跳到第8题 skip to Q8<br>3 = 孕妇 Pregnant woman → 跳过本单元 skip this section	
1. 宝宝出生体重 Child's weight at birth<br><br>【优先根据出生证明填写】 Ask respondent to read information from baby's birth certificate.	克grams
2. 宝宝出生身长 Child's birth length<br><br>【优先根据出生证明填写】Ask respondent to read information from baby's birth certificate.	厘米cm
3. 你在哪里生的宝宝？Where did you give birth?	1=村诊所 village clinic<br>2=私人诊所 Private clinic<br>3=乡镇卫生院 Township Health Center<br>4=县妇幼保健院 County Maternal and Child Health Hospital<br>5=县医院 County Hospital<br>6=市妇幼保健院 City Maternal and Child Health Hospital<br>7=市医院 City Hospital<br>8=职工医院 staff hospital<br>9=其他，请注明Other, please specify _____
4. 宝宝是第多少孕周出生的？At what gestational age was the baby born?	____周week

【孕周取整数（向下取整，比如22周6天，算作22周）】Round down: for example, for 22 weeks and 6 days record 22 weeks.<br><br>【优先根据出生证明填写】Ask respondent to read information from baby's birth certificate.	
5. 宝宝生产方式？How was the child delivered?<br><br>【如果不清楚是否使用助产钳或吸引器，就选自然分娩】If respondent is unclear how child was delivered, select natural birth.	1=自然分娩 natural vaginal Birth<br>2=辅助分娩（使用助产钳或吸引器）assisted vaginal birth (used forceps and/or suction)<br>3=剖宫（腹）产C-Section<br>9=不知道Don't know
6. 宝宝出院后接受过多少次体检（包括在家或者医院）？How many check-ups did your child have in total after being discharged from hospital (at home or clinics)?	___次 times
7. 宝宝出院后10天内有没有做过体检，或者接受医务人员上门检查？ Did the baby go to a well-baby visit or receive a health check-up home visit from a doctor within the first 10 days after being discharged?<br><br>【宝宝未满10天，就截止目前为止】 If younger than 10 days, ask "up till now"	1=是yes<br>2=否no<br>999= If doesn't know 不知道
8. 宝宝最近一次接受体检是什么时候？ When was the most recent check-up?	___年 year<br>____月 month<br>____日day<br><br>999 if Don't know 不知道
9. 你的宝宝打过几次疫苗了？How many times has child been vaccinated? （0 = 0 time -> skip to Q12， 999= Don't know 不知道 -> skip to Q11）	____次
10. 宝宝每次打疫苗时多大？ How old was your child each time he/she was vaccinated?<br><br>【依次问第一次、第二次、直到第n次（创建二维表）】 Ask about the age of the baby's vaccination, starting from 1st, 2nd, until n-th vaccination	____年<br>____月<br>____天<br><br>999 if Don't know 不知道
11. 宝宝打疫苗付过钱吗？ Did you ever pay for any vaccinations?	0=没有 no<br>1=付过 yes<br>999=不知道 unknown

12. 你估计你们镇上两岁以下的小孩有百分之多少按时完成了应该打的疫苗？【回答一个百分数】 What percent of children under 2 years in your town do you think are up-to-date on their vaccinations?	_____ %<br><br>999=不知道 unknown

C2. 妈妈/孕妇保健 Perinatal Care

**核实是否为目标样本：目标样本是孕妇或者11月12号后出生的宝宝妈妈吗？**<br>**ELIGIBILITY CHECK: Is the respondent a pregnant woman or mother of babies born after 2019-11-12?**<br>1=孕妇 Pregnant woman → 回答第1-4题<br>2=宝宝生于11月12号之后的妈妈（基线调研之后出生） mother of baby born after 2019-11-12 → 回答所有题 answer all questions<br>3=以上都不是 → 不做本单元 skip this section	
1. 在您怀孕期间，医务人员是否到过您家家访？During your pregnancy, did any medical provider visit you at home?	1=是Yes<br>2=否No
2. 怀着这个宝宝的时候（妈妈）是否进行过产前检查？When you were pregnant, have you had any prenatal visit at the hospital/clinic?	1=yes是<br>2=no 否→ skip to Q5<br>跳至5题
3. 您总共进行了几次产前检查？How many prenatal visits did you have altogether?	____次times
4 您最近一次产前检查是什么时候？ When was your last prenatal visit?<br><br>【注意：孕妇和基线调研之后出生的宝宝都需要问此问题】	___年 year<br>____月 month<br>____日day
5. 在您生完孩子出院后，医务人员是否给您做过体检？Did any medical staff give you a medical examination after you gave birth and left the hospital?	1=是Yes<br>2=否No → 结束本单元 end of section
6. 您在哪些地点进行过体检？ where did the exam take place?<br><br>【多选】 [multiple choice]	1=在家at home<br>2=县级医院及县级以上医院 county-level hospital or above<br>3=镇级医院township-level hospital<br>4=村诊所village clinic<br>5=其它，注明other, specify_____

C3. 影响婴幼儿健康的行为 Behaviors Influencing Child Health

**核实是否是目标样本：该样本是宝宝吗？**<br>**ELIGIBILITY CHECK: Is the index a baby ?**<br>1=是yes → 回答全部 all questions<br>2=否no → 跳过本单元 skip this section

**1.** 回忆一下，您昨天什么时候洗手了Think about how you spent your day yesterday. When did you wash your hands?<br>【不要读选项，可回答多项】**Do not read answer options**; choose all that apply	1=早上第一件事就是洗手First thing in the morning<br>2=睡觉前Right before going to sleep<br>3=做饭前Before cooking<br>4=做饭后After cooking<br>5=吃饭前Before eating<br>6=吃饭后After eating<br>7=小便后After peeing<br>8=大便后After pooing<br>9=手看上去脏的时候When hands have visible dirt on them<br>10=照顾宝宝前before handling the baby<br>11=照顾宝宝后after handling the baby<br>12=给宝宝换尿布后after changing the baby's diapers<br>13=给宝宝擦屁股之后<br>14=咳嗽之后 coughing<br>15= 打喷嚏之后 sneezing<br>16= 其它，请注明Other, specify:_____
2. 给宝宝准备或喂食物之前您洗手的频率？How frequently do you wash your hands when you feed your baby?	1=从不Never<br>2=很少Rarely<br>3=经常Often<br>4=总是Always

D. 喂养行为 Nutrition and Feeding Behavior

D1. 婴幼儿喂养习惯 Breastfeeding and IYCF Practice

**核实目标样本：该样本是宝宝吗？**<br>**ELIGIBILITY CHECK: Is the index a baby?**<br>1=2019年11月12日以后出生的宝宝 Baby born after 2019-11-12 -> 回答全部问题answer all questions<br>2=2019年11月12日以前出生的宝宝 Baby born before 2019-11-12 -> Starts from Q8 从第8题起<br>3=no 不是→ skip this section 跳过本部分

1. 这个宝宝喝过母乳吗？Has the child ever been breastfed?	1=是yes<br>2=否no
2. 宝宝出生后多久第一次吮吸乳头？How soon after birth did the baby suckle at the breast for the first time?<br>【如果少于1小时，圈出“1”，并记录00小时；<br>如果少于24小时，圈出“1”并记录小时数，从01到23；<br>如果大于24小时，圈“2”并记录完成的天数。】<br>[If respondent reports she put the infant to the breast in less than 1 hour, circle ‘1’ for hours AND RECORD ‘00’ hours. If less than 24 hours, circle ‘1’ and record number of completed hours, from 01 to 23. Otherwise, circle ‘2’ and record number of completed days.]	1=小时hour |__|__| 2=天days |__|__| 3=从来没有吮吸过乳头never<br>999=不知道don’t know
3. What was the child fed first after birth?<br>宝宝出生后第一口喂的是什么？**【不要读选项】**Do not read response options	1=母乳/初乳breast milk/colostrum<br>2=配方奶formula<br>3=水water<br>4=其它（注明）other (specify)______<br>999=不知道don’t know
4. 是否给宝宝喂了初乳（初乳是产后一周内产生的淡黄色、粘稠的母乳）Was the child fed colostrum?<br><br>【初乳是产后一周内产生的淡黄色、粘稠的母乳】	1=是yes<br>2=否no<br>999=不知道don’t know
5. 宝宝在医院的时候被喂过水吗？Was the baby fed water at any time in the hospital?	1=是yes<br>2=否no<br>999=不知道don’t know
6. 宝宝在医院的时候被喂过奶粉吗？Was the baby fed formula at any time in the hospital?	1=是yes<br>2=否no<br>999=不知道don’t know
7. 宝宝在医院的时候被喂过糖水吗？Was the baby fed sugared water at any time in the hospital?	1=是yes<br>2=否no<br>999=不知道don’t know
8. Was the child breastfed yesterday during the day or at night?<br>昨天（包括白天和晚上）宝宝是否喝过母乳？	1=是yes<br>2=否no → 第10题 skip to Q10<br>999=不知道don’t know→第10题 skip to Q10
9. 宝宝昨天（包括白天和晚上）喝了几次母乳？How many times yesterday during the day or at night was the child breastfed?	___次times<br>999=不知道don’t know

10. 宝宝昨天（包括白天和晚上）有没有喝水？Did the child have any plain water yesterday?	1=是yes<br>2=否no<br>999=不知道don't know
11. Did the child have any infant formula yesterday?<br>宝宝昨天（包括白天和晚上）有没有喝婴儿配方奶粉？	1=是yes<br>2=否no → 第14题 skip to 14<br>999=不知道don't know→第14题 skip to 14
12. 宝宝昨天（包括白天和晚上）喝了几次配方奶粉？How many times yesterday did the child consume infant formula?	___次times<br>999=不知道don't know
13. 宝宝昨天（包括白天和晚上）喝了多少毫升的配方奶粉？How many milliliters of infant formula did the child consume yesterday?	___毫升ML<br>999=不知道don't know
14. 什么时候开始给宝宝喂配方奶粉? At what age did the baby start to have formula?	______月month<br>0=从出生就开始喝配方奶 from birth<br>888=未添加奶粉has not started
15. 您多久洗一次宝宝的奶瓶？How often do you wash your baby's bottles?<br>【不要读选项】Do not read response options	0=没有/不用奶瓶Don't have/don't use bottles → 跳到17题skip to Q17<br>1=每次用完就洗after every use<br>2=每天Every day<br>3=每周Every week<br>4=每两周Every two weeks<br>5=每个月Every month<br>6=少于每个月一次Less than once a month<br>7=从来不洗Never →跳到17题 skip to Q17
16. 通常，您怎么给宝宝洗奶瓶How do you wash your baby's bottles?<br>【不要读选项】Do not read response options	1=只用冷水冲洗Rinse with cold water<br>2=用开水煮或者烫boil or rinse with boiling water<br>3=用特制洗奶瓶器Use a designated bottle-sanitizing device<br>4=用洗洁精和水洗Wash with soap and water<br>6=其它，请注明Other, specify:_______

17. Did the child have any milk such as tinned, powdered, or fresh animal milk yesterday? 宝宝昨天（包括白天和晚上）有没有喝新鲜动物奶（比如牛奶、羊奶）或动物奶粉？	1=是yes<br>2=否no → skip to 19<br>999=不知道don't know→ skip to 19
18. 宝宝昨天（包括白天和晚上）喝了几次新鲜动物奶或动物奶粉？How many times yesterday did the child consume any milk of the kinds just asked about?	___次times<br>999=不知道don't know
19. 宝宝昨天（包括白天和晚上）有没有喝过果汁或果汁饮料？ Did the child have any juice or juice drink yesterday?	1=是yes<br>2=否no<br>999=不知道don't know
20. 宝宝昨天（包括白天和晚上）有没有喝过汤？Did the child have any clear broth yesterday?	1=是yes<br>2=否no<br>999=不知道don't know
21. 宝宝昨天（包括白天和晚上）有没有喝酸奶？Did the child have any yogurt yesterday?	1=是yes<br>2=否no → skip to Q23<br>999=不知道don't know → skip to Q23
22. 宝宝昨天（包括白天和晚上）有没有喝粥？Did the child have any thin porridge yesterday?	1=是yes<br>2=否no<br>999=不知道don't know
23. 宝宝昨天（包括白天和晚上）有没有喝益生菌乳品饮料，比如养乐多？Did the child have any probiotic dairy drink such as Yakult yesterday?	1=是yes<br>2=否no<br>999=不知道don't know
24. 宝宝昨天（包括白天和晚上）有没有喝其他饮品，比如汽水、茶、豆浆等Did the child have any other liquid yesterday (e.g., sugar water, soda, tea, soy milk)?	1=是yes<br>2=否no<br>999=不知道don't know
25. 宝宝是开始添加辅食了吗（除了母乳和配方奶之外给宝宝提供主要能量来源的液体、半固体、固体食物）？ Has the baby started to have complementary food (liquid, semi-solid, or solid foods other than breastmilk or formula that provide nutrients)	1=Yes 是<br>**2=No 否 → End of Section 结束本部分**
26. 宝宝是在几月龄开始添加辅食（除了母乳和配方奶之外给宝宝提供主要能量来源的液体、半固体、固体食物）？ At what age did the baby start to have complementary foods (liquid, semi-solid, or solid foods other than breastmilk or formula that provide nutrients)?	______月month<br><br>999=不知道don't know
27. 宝宝昨天是否吃了米汤、粥、面汤、馒头或米饭等主食类食物？Did the baby eat any staple food yesterday, such as rice porridge, flour porridge, steamed bun or rice	1=是yes<br>2=否no<br>999=不知道don't know

28. 宝宝昨天是否吃了南瓜、胡萝卜、红心红薯等里面是黄色或橙色的食物? Did the baby eat any yellow or orange vegetables yesterday, such as pumpkin, carrot or red sweet potato<br><br>【“黄色或红色”指把食物切开后里面的颜色】”Yellow/orange” refers to color of the inside of the vegetables.	1=是yes<br>2=否no<br>999=不知道don’t know
29. 宝宝昨天是否吃了土豆、山药、白萝 卜、白心红薯等根茎类食物? Did the baby eat any root and stem vegetables yesterday, such as potato, yam, radish, white sweet potato	1=是yes<br>2=否no<br>999=不知道don’t know
30. 宝宝昨天是否吃了深绿色叶子菜? （举例：菠菜、豌豆尖） Did the baby eat any leafy dark green vegetables yesterday?	1=是yes<br>2=否no<br>999=不知道don’t know
31. 宝宝昨天是否吃了红色或黄色的水果，如柿子、杏、西瓜、哈密瓜或番茄? Did the baby eat any red or yellow fruits such as persimmon, apricot, watermelon, cantaloupe or tomato yesterday?<br><br>【“红色或黄色”指把食物切开后里面的颜色】”Yellow/orange” refers to color of the inside of the fruits.	1=是yes<br>2=否no<br>999=不知道don’t know
32. 宝宝昨天是否吃了其他水果或蔬菜? Did the baby eat any other fruits or vegetables yesterday?	1=是yes<br>2=否no<br>999=不知道don’t know
33. 宝宝昨天是否吃了内脏类食物，如动物肝、肾或心脏? Did the baby eat any organ meats such as animal liver, kidney or heart yesterday?	1=是yes<br>2=否no<br>999=不知道don’t know
34. 宝宝昨天是否吃了肉类或肉类产品（如鸡肉、鸭肉、猪肉、牛肉、羊肉等）? Did the baby eat any other meat or meat products (e.g., chicken, duck, pork, beef, lamb, etc.) yesterday?	1=是yes<br>2=否no<br>999=不知道don’t know
34. 宝宝昨天是否吃了鸡蛋? Did the baby eat any eggs yesterday?	1=是yes<br>2=否no<br>999=不知道don’t know
36. 宝宝昨天是否吃了鱼类、贝类或海鲜类食物? Did the baby eat any fresh or dried fish, shellfish or seafood yesterday?	1=是yes<br>2=否no<br>999=不知道don’t know
37. 宝宝昨天是否吃了扁豆等豆类、豆制品或坚果、种子? 注意：喝过豆浆算吃过豆制品? Did the baby eat any beans, peas, lentils, nuts or seeds yesterday? Please note: Drinking soymilk counts	1=是yes<br>2=否no<br>999=不知道don’t know
38. 宝宝昨天是否吃了乳制品，如奶酪和酸奶? Did the baby eat any dairy products such as cheese and yoghurt yesterday?	1=是yes<br>2=否no<br>999=不知道don’t know

F. 新冠肺炎影响 COVID-19

F1. 新冠肺炎风险感知 COVID-19 Risk Perception

(接下来我将和您聊一下新冠疫情爆发的封村期间，您的一些感受与行为；Next, I will talk to you about some of your feelings and behaviors during the shutdown of the village.）

**1.** 您了解怎么预防新冠肺炎吗？<br>Did you know how to prevent COVID-19?	1=完全不了解；Nothing<br>2=比较不了解；Less<br>3=一般；Neutral<br>4=比较了解；More<br>5=完全了解All
**2.** 您觉得您家人了解怎么预防新冠肺炎吗？<br>Did you think your family members know how to prevent COVID-19?	1=完全不了解；Nothing<br>2=比较不了解；Less<br>3=一般；Neutral<br>4=比较了解；More<br>5=完全了解All
**3.** 您觉得自己得新冠肺炎的可能性有多大？<br>What was the probability of getting infected with COVID-19 to you?	1=根本不会；Not at all<br>2=可能不会；Probably not<br>3=一般；Neutral<br>4=可能会；Probably<br>5=肯定会Definitely
**4.** 您觉得您家人得新冠肺炎的可能性有多大？<br>What was the probability of getting infected with COVID-19 to your family members?	1=根本不会；Not at all<br>2=可能不会；Probably not<br>3=一般；Neutral<br>4=可能会；Probably<br>5=肯定会Definitely
5. 您觉得您村里/镇上的人得新冠肺炎的可能性有多大？What was the probability of getting infected with COVID-19 to villagers or townsfolk?	1=根本不会；Not at all<br>2=可能不会；Probably not<br>3=一般；Neutral<br>4=可能会；Probably<br>5=肯定会Definitely
**6.** 您觉得您做的个人防护能保护自己不得新冠肺炎吗？	1=根本保护不了；Not at all<br>2=可能保护不了；Probably not<br>3=一般；Neutral<br>4=比较能保护；Probably

Did you think that your personal precaution can protect you from getting infected with COVID-19?	5=完全能保护Definitely
**7.** 您觉得您家人做的防护措施能保护他们不得新冠肺炎吗？<br>Did you think that your family members' precaution can protect them from getting infected with COVID-19?	1=根本保护不了；Not at all<br>2=可能保护不了；Probably not<br>3=一般；Neutral<br>4=比较能保护；Probably<br>5=完全能保护Definitely
**8.** 您觉得村里/乡镇做的防护措施能保护大家不得新冠肺炎吗？<br>Did you think that village or township officials' precaution can protect people from getting infected with COVID-19?	1=根本保护不了；Not at all<br>2=可能保护不了；Probably not<br>3=一般；Neutral<br>4=比较能保护；Probably<br>5=完全能保护Definitely
9. 在您做防护的时候，如果遇到一些问题（比如买不到口罩及洗手液等、用起来不方便、家人不支持等），您相信自己能很好地解决吗？<br>When taking some precaution measures, if you encounter some problems (such as not being able to buy protective items, inconvenient to use, unsupported by your family members, etc.), do you believe you can solve it well?	1=非常不相信；Mostly unbelieve<br>2=比较不相信；Less believe<br>3=一般；Neutral<br>4=比较相信；More believe<br>5=非常相信 Mostly believe
10. 新冠疫情让您感到害怕吗？ Did the epidemic of COVID-19 make you scared?	1=完全不害怕；Not scared at all<br>2=比较不害怕；Less scared<br>3=一般；Neutral<br>4=比较害怕；More scared<br>5=非常害怕 Very scared
11. 新冠疫情让您感到焦虑吗？ Did the epidemic of COVID-19 make you anxious?	1=完全不焦虑；Not anxious at all<br>2=比较不焦虑；Less anxious<br>3=一般；Neutral<br>4=比较焦虑；More anxious<br>5=非常焦虑；Very anxious
**12.** 假如得了新冠肺炎的话，您觉得会致命吗？<br>If you were infected with COVID-19, did you think that it was very fatal for you？	1=根本不会；Not at all<br>2=可能不会；Probably not<br>3=一般；Neutral<br>4=可能会；Probably<br>5=肯定会Definitely
13. 假如您的家人得了新冠肺炎的话，您觉得对于	1=根本不会；Not at all

他们来说会致命吗？<br>If your family members were infected with COVID-19, did you think that it was very fatal for them？	2=可能不会；Probably not<br>3=一般；Neutral<br>4=可能会；Probably<br>5=肯定会Definitely
14. 您最主要是从哪里知道新冠肺炎的？（单选）<br>Where did you get the information related to COVID-19?	1= 村/乡镇的宣传（如广播、宣传单、海报、黑板报等）; Village / township propaganda (such as radio, leaflets, posters, blackboard newspapers, etc.)<br>2=电视; TV<br>3=手机短信/打电话; Phone message /phone call<br>4=微信/QQ/微博/抖音等网络新媒体平台; New media platforms such as WeChat / QQ / Blog / Tik Tok, etc;<br>5=家人、亲友、街邻面对面；Face to face with family members, relatives and friends
15. 您觉得上述最主要的信息来源可信吗？ Do you think the main source you get the information related to COVID-19 above is trustworthy?	1=非常不可信Mostly untrusted<br>2=不太可信；Less trusted<br>3=一般；Neutral<br>4=比较可信；More trusted<br>5=非常可信；Mostly trusted
16. 您最关注的新冠肺炎相关的内容是什么？（单选）<br>What was your main focus on the COVID-19?	1=村里患病情况；Prevalence information in the village<br>2= 乡/镇里的患病情况; Prevalence information in the township<br>3=县/区级及以上的患病情况; Prevalence information in county level and above<br>4=村里的治愈情况；Cure information in the village<br>5=乡/镇里的治愈情况; Cure information in the township<br>6=县/区级及以上的治愈情况; Cure information in county level and above<br>7=村里的病死情况; Death information in the village<br>8=乡/镇里的病死情况; Death information in the township<br>9=县/区级及以上的病死情况; Death information in county level and above<br>10=政府的管控和防护政策；Government control and precaution policies<br>11=口罩、消毒液等个人防护产品的供应信息; Supply information of personal protective products, such as masks and disinfectants, etc;<br>12=日用品、食品等生活物资的供应信息; Supply information of daily necessities, such as food and other living materials, etc.<br>13=其他，请说明:____________; other, please explain______________________

17. **封村期间，**您出家门会戴口罩吗？ During the village shutdown, did you wear mask when you go outside?	0= 没有出过门；Never go outside<br>1=每次都不会；Never<br>2=经常不会；Not often<br>3=偶尔会；Sometimes<br>4=经常会；Often<br>5=每次都会Every time
18. **封村期间，**您回到家里会用洗手液/香皂/肥皂洗手吗？<br>During the village shutdown, did you wash hands using the soap or detergent (bar soap, liquid soap, detergent, etc.) when you came back home?	0= 没有出过门；Never go outside<br>1=每次都不会；Never<br>2=经常不会；Not often<br>3=偶尔会；Sometimes<br>4=经常会；Often<br>5=每次都会Every time
**19. 封村期间，**如果听说什么能防治新冠肺炎，您就会想办法去买吗？<br>During the village shutdown, did you try to buy anything which were recommended to prevent or cure the COVID-19 once you heard about it?	1=完全不符合；Strongly disagree<br>2=比较不符合；Mildly disagree<br>3=一般；Neutral<br>4=比较符合；Mildly agree<br>5=完全符合Strongly agree
**20. 封村期间，**您有没有一看（听）到疫情相关信息就转发（转告）给他人？<br>During the village shutdown, did you forward to others any messages related to the COVID-19 as soon as you saw them?	1=完全没有；Not at all<br>2=基本没有；Almost not;<br>3=偶尔有；Sometimes<br>4=经常有；Often<br>5=总是有Always
**21. 封村期间，**您有没有排斥所有外村/乡镇的人？<br>During the village shutdown, did you exclude all outsiders from other villages or townships?	1=完全没有；Not at all<br>2=基本没有；Almost not;<br>3=偶尔有；Sometimes<br>4=经常有；Often<br>5=总是有Always

F2. 新冠肺炎症状与感染COVID-19 Symptoms and infections

1. Have you or anyone in your household had the following symptoms since January?<br>自从今年1月以来过去的几个月中您或者您的家人有没有以下的的症状？<br><br>【多选】[select all that apply]	0 = No 没有<br>1 = Fever 发烧<br>2 = Cough 咳嗽<br>3 = Shortness of breath 气短<br>4 = Chills; repeated shaking with chills 发抖/发冷<br>5 = Muscle pain 肌肉酸痛<br>6 = Headache 头疼

	7 = Sore throat 嗓子疼<br>8 = Loss of taste or smell 味觉或嗅觉丧失<br>9 = Other, please specify 其他，请注明：_______
2. Has anyone from your household been tested for COVID-19 virus since January?<br>自从今年1月以来您或者您的家人有没有人做过新冠肺炎的检测？	1 = Yes 有<br>2 = No 没有 → skip to Q4
3. Has anyone in your household tested positive for COVID-19?<br>在您或者您的家人中，有没有人确诊新冠肺炎？	1 = Yes 有<br>2 = No 没有
4. Do you know anyone outside of your household who has tested positive for COVID-19?<br>除了您家人以外，您认识其他确诊了新冠肺炎的人吗？	1 = Yes 有<br>2 = No 没有

G. 母亲心理健康 Maternal Mental Health

**核实是否为目标样本：目标样本是孕妇或者宝宝的妈妈吗？**<br>**ELIGIBILITY CHECK: Is the respondent a pregnant woman or mother of children?**<br>1. Yes 是<br>2. No 否 -> skip to next section 跳到下一部分	
你正怀着宝宝或者最近生了宝宝，我们想了解一下您的感受，请根据您过去七天的感受选择答案，而不仅仅是今天。As you are pregnant or have recently had a child, we would like to know how you are feeling. Please choose the answer that comes closest to how you have felt IN THE PAST 7 DAYS, not just how you feel today.	
在过去七天 In the past 7 days …<br><br>【读条目，**不要解释**，如果被访者听不明白，再读一遍】<br>Read the statements. **Do not explain the statement in your own words.** If the respondent does not understand the statement, read the statement again.	选项 Options
1. 我能看到事情有趣的一面，并笑得开心。 I have been able to laugh and see the funny side of things as much as I always could.	0=和以前一样As much as I always could<br>1=比以前少一些Not quite so much now.<br>2=肯定比以前少 Definitely not so much now<br>3=完全做不到 Not at all

2. 我对未来有所期待。 I have looked forward with enjoyment to things.	0=和以前一样 As much as I ever did<br>1=比以前少一些Rather less than I used to<br>2=肯定比以前少Definitely less than I used to<br>3=完全做不到Hardly at all
3. 当事情出错时，我会过分得责备自己 I have blamed myself unnecessarily when things went wrong.	3=大部分时候这样Yes, most of the time.<br>2=有時候这样Yes, some of the time<br>1=不经常这样Not very often<br>0=沒有这样No, never
4. 我会无缘无故感到焦虑和担心 I have been anxious or worried for no good reasons.	0=一点也沒有No, not at all.<br>1=极少有Hardly, ever<br>2=有时候这样 Yes, sometimes<br>3=经常这样Yes, very often
5. 我无缘无故感到害怕和惊慌 I have felt scared or panicky for no very good reason.	3=大部分时候这样样Yes, quite a lot<br>2=有时候这样Yes, sometimes<br>1=不经常这样No, not much<br>0=沒有这样No, not at all
6. 当很多事情冲着我来，使我透不过气Things have been getting on top of me.	3=大多数时候不能应付 Yes, most of the time I haven't been able to cope at all<br>2=有时不能应付Yes, sometimes I haven't been coping as well as usual<br>1=基本可以应付No, most of the time I have coped quite will<br>0=一直可以应付No, I have been coping as well as ever
7. 我很不开心，以致失眠 I have been so unhappy that I have had difficulty sleeping	3=经常这样Yes, most of the time<br>2=有时这样Yes, sometimes<br>1=偶尔这样Not very often<br>0=沒有这样No, not at all
8. 我感到难过或悲伤 I have felt sad or miserable.	3=经常这样Yes, most of the time<br>2=有时这样Yes, quite often<br>1=偶尔这样Not very often<br>0=沒有这样No, not at all
9. 我不开心到哭 I have been so unhappy that I have been crying.	3=经常这样Yes, most of the time<br>2=有时这样Yes, quite often<br>1=偶尔这样Not very often<br>0=沒有这样No, not at all

10. 我想过要伤害自己 The thought of harming myself has occurred to me.	3=经常这样 Yes, quite often<br>2=有时候这样Sometimes<br>1=很少这样 Hardly ever<br>0=从来沒有Never

H. 开销 Expenditures

**核实是否为目标样本：该样本是宝宝？**<br>**ELIGIBILITY CHECK: Is the index a baby?**<br>1=是 Yes<br>2=否 No → 不做本单元skip this section	
1. How much did you and your family spend on snacks for your child in the past month? 您家最近一个月给宝宝购买零食大概花费了多少钱？	____ 元 RMB
2. How much did your family spend on food in the past month? 您家最近一个月买菜大概花费了多少钱？	____ 元 RMB
3. How much did you and your family spend on micronutrient supplements for your child in the past month? 您家最近一个月给宝宝购买微量元素补充剂大概花费了多少	____ 元 RMB
4. How much did you and your family spend on alcohol and cigarettes in the past month? 您家最近一个月买烟酒大概花了多少钱？	____ 元 RMB
5. How much did you and your family spend on gifts for friends and family in the past month? 您家最近一个月给亲戚朋友送礼大概花了多少钱？	____ 元 RMB
6. How many cans of formula did your child consume in the past month? 您孩子最近一个月喝了多少罐奶粉？	___ units 罐<br>如果0罐奶粉if 0, 结束本单元→ end of section
7. What was the price for the formula you bought last time? 您上次购买的奶粉每罐多少钱？	____ 元 RMB
8. How much did you spend on formula in the past month? 您上个月给孩子买奶粉一共花了多少钱？	____ 元 RMB
9. 你们地区是什么时候解封的？ When was the reopening date in your area?<br><br>【备注：可填写大概日期】An estimated date is allowed.	___月 month<br>___日 day<br><br>999 如果不知道解封日期if the specific date is unclear
10. Before reopening, was there anyone in your household who was unable to work because of the COVID outbreak? (e.g., workplace closed, cannot travel; infected by virus) ？<br>今年解封之前，您家里有没有人因为病毒影响不能工作？(例如，打工/工作的地方停工，没有交通，被感染新冠）	1=Yes 有<br>2=No 没有

11. Before reopening, was there anyone in your household who was able to work, but chose not to because they were afraid of being infected?<br>今年解封之前，您家里有没有人本来有工作可以做，但因为担心病毒感染，选择不出去工作？	1=Yes 有<br>2=No 没有
12. Since reopening, is there anyone in your household who has been unable to work because of the COVID outbreak? (e.g., workplace closed, cannot travel; infected by virus) 自解封之后，您家里有没有人因为病毒影响不能工作？(例如，打工/工作的地方停工，没有交通，被感染新冠）？	1=Yes 有<br>2=No 没有
13. Since reopening, is there anyone in your household who has been able to work, but has chosen not to because they were afraid of being infected?<br>自解封之后，您家里有没有人本来有工作可以做，但因为担心病毒感染，选择不出去工作 ？	1=Yes 有<br>2=No 没有
